# Racial and Ethnic Residential Segregation and Monocyte DNA Methylation Age Acceleration

**DOI:** 10.1001/jamanetworkopen.2023.44722

**Published:** 2023-11-29

**Authors:** Margaret T. Hicken, John Dou, Kiarri N. Kershaw, Yongmei Liu, Anjum Hajat, Kelly M. Bakulski

**Affiliations:** 1 Institute for Social Research, University of Michigan, Ann Arbor; 2 Department of Epidemiology, School of Public Health, University of Michigan, Ann Arbor; 3 Department of Preventive Medicine, Northwestern University, Chicago, Illinois; 4 Department of Medicine, Duke University, Durham, North Carolina; 5 Department of Epidemiology, University of Washington, Seattle

## Abstract

**Importance:**

Neighborhood segregation and poverty may be important drivers of health inequities. Epigenomic factors, including DNA methylation clocks that may mark underlying biological aging, have been implicated in the link between social factors and health.

**Objective:**

To examine the associations of neighborhood segregation and poverty with 4 DNA methylation clocks trained to capture either chronological age or physiological dysregulation.

**Design, Setting, and Participants:**

This cohort study uses data from the Multi-Ethnic Study of Atherosclerosis (MESA), a longitudinal study that started in 2000 to 2002, with follow-up in 2002 to 2004, 2004 to 2005, 2005 to 2007, and 2010 to 2012. In 2000 to 2002, adults who identified as White or Black race or Hispanic or Chinese ethnicity in 6 US sites (Baltimore, Maryland; Chicago, Illinois; Forsyth County, North Carolina; Los Angeles County, California; Northern Manhattan, New York; and St. Paul, Minnesota) were sampled for recruitment. A random subsample of 4 sites (Maryland, North Carolina, New York, and Minnesota) were selected for inclusion in the MESA epigenomics ancillary study at examination 5 (2010-2012). Participants who identified as White or Black race or Hispanic ethnicity, were aged 45 to 84 years, and did not have clinical cardiovascular disease were included in this analysis. Data were analyzed from May 2021 to October 2023.

**Exposure:**

Information on 2000 census tract poverty and Getis-Ord *G* statistic segregation of Hispanic residents, non-Hispanic Black residents, or non-Hispanic White residents were linked to participant addresses at examination 1 (2000-2002).

**Main Outcomes and Measures:**

At examination 5, DNA methylation was measured in purified monocytes. DNA methylation age acceleration was calculated using 4 clocks trained on either chronological age or physiological dysregulation. Linear regressions were used to test associations.

**Results:**

A total of 1102 participants (mean [SD] age, 69.7 [9.4] years; 562 [51%] women) were included, with 348 Hispanic participants, 222 non-Hispanic Black participants, and 533 non-Hispanic White participants. For non-Hispanic Black participants, living in tracts with greater segregation of Black residents was associated with GrimAge DNA methylation age acceleration, a clock designed to capture physiological dysregulation. A 1-SD increase in segregation was associated with 0.42 (95% CI, 0.20-0.64) years age acceleration (*P* < .001); this association was not observed with other clocks. This association was particularly pronounced for participants living in high poverty tracts (interaction term, 0.24; 95% CI, 0.07-0.42; *P* = .006). In the overall sample, census tract poverty level was associated with GrimAge DNA methylation age acceleration (β = 0.45; 95% CI, 0.20-0.71; adjusted *P* = .005).

**Conclusions and Relevance:**

These findings suggest that epigenomic mechanisms may play a role in the associations of segregated and poor neighborhoods with chronic conditions.

## Key Points

**Question:**

Are neighborhood segregation and poverty associated with composite measures of DNA methylation thought to reflect biological age (ie, DNA methylation age acceleration clocks)?

**Findings:**

In this cohort study of 1102 racially and ethnically diverse adults ages 55 to 94 years, a 1-SD increase in residential segregation in 2000 to 2002 was associated with 0.41 years biological age acceleration in 2010, as captured by the GrimAge DNA methylation age acceleration clock (a clock trained to capture methylation sites related to physiologic dysregulation) for non-Hispanic Black participants.

**Meaning:**

These findings suggest that neighborhood social context may be associated with an underlying biological age acceleration at the epigenomic level.

## Teaser

This cohort study examines associations of residential racial and ethnic segregation and census tract–level poverty with DNA methylation age using 4 DNA methylation clocks.

## Introduction

Racial and ethnic inequities in healthy aging are well-known, with Black adults experiencing greater risk and earlier onset of chronic conditions, such as cardiovascular disease (CVD), hypertension, and diabetes, compared with White adults.^1^ Neighborhood context has emerged as a potentially powerful determinant of racial and ethnic health inequities and may be a key intervention site.^2,3,4,5,6,7^ Evidence indicates the stark racial and ethnic inequities in exposure to segregated, underresourced, oversurveilled, and polluted neighborhoods.^8,9,10,11,12,13,14,15,16,17,18^ Racial and ethnic residential segregation may be a fundamental mechanism by which inequalities are maintained through differential access resources and differential exposures to social stressors and environmental hazards.^11,12,16,19,20,21,22,23,24,25,26,27^ Changes in neighborhood resources and segregation levels have been associated with improvements in CVD risk factors.^26,27,28^

Research on neighborhoods and health generally focuses on specific health outcomes,^29,30,31,32,33,34,35,36^ which may underestimate the overall impact of racially and ethnically unequal neighborhood exposures.^37^ The literature suggests the importance of epigenetic factors, particularly DNA methylation, linking socioenvironmental context to health.^38,39,40,41,42,43,44,45,46^ Epigenetic factors are changes to DNA structure outside of the DNA sequence that result in changes in gene activity or function. DNA methylation is the most well-characterized epigenetic factor sensitive to socioenvironmental factors.^39,40,41,42,43,44,45,46^

In the nascent social epigenomics field, reports suggest that neighborhood exposures may be associated with DNA methylation patterns, although the associations are inconsistent.^47,48,49^ For example, neighborhood poverty, but not neighborhood income or education, was a marker associated with global DNA methylation.^50^ Other studies have reported that indices of neighborhood social disadvantage and social stress were related to the methylation of certain tumor suppressor-, stress-, and inflammation-related genes.^39,51,52^

Recent work has focused on DNA methylation age clocks (hereafter, *clocks*), which are weighted means of methylation at multiple sites.^53^ The methylation sites selected for inclusion in clock algorithms are generally determined based on their association with an outcome (eg, chronological age). Some are designed to estimate chronological age (eg, Horvath clock,^54^ Hannum clock^55^). Others are designed to assess physiologic dysfunction (eg, PhenoAge clock,^56^ GrimAge clock^57^). As with the older DNA methylation studies, studies reporting on associations of neighborhood social context with these clocks have been inconsistent. In a sample of adults in Detroit, Michigan, neighborhood poverty was not associated with the Hannum, Horvath, or PhenoAge clocks.^58^ On the other hand, a neighborhood deprivation index was associated with the Hannum, GrimAge, and PhenoAge clocks but not the Horvath clock.^59^

To date, there has been little research on these clocks beyond their associations with neighborhood socioeconomic characteristics. While residential segregation and socioeconomic deprivation are correlated, there is racial and ethnic variation. In the US, middle-class Black individuals often live in segregated neighborhoods of higher poverty compared with middle-class White individuals.^60^ Neighborhood poverty and segregation may operate together to result in particularly severe disinvestment.^61,62^ Our objective is to examine the independent and interactive associations of neighborhood racial and ethnic segregation and poverty with later DNA methylation age acceleration (DMAA) using 4 clocks trained to capture either chronological age or physiological dysregulation, in a racially and ethnically diverse sample of adults. We hypothesize that living in neighborhoods characterized by the segregation of non-White residents (ie, Hispanic and non-Hispanic Black residents) and/or high poverty will be associated with markers of accelerated aging compared with living in other neighborhoods.

## Methods

This cohort study was a secondary analysis of data from the Multi-Ethnic Study of Atherosclerosis (MESA) and was approved by the University of Michigan institutional review board. All participants provided written informed consent at the time of participation. This report follows the Strengthening the Reporting of Observational Studies in Epidemiology (STROBE) reporting guideline for cross-sectional observational studies. More details are provided in the eMethods in Supplement 1.

### Data Set

The MESA is a US prospective cohort study conducted at 6 sites: Baltimore, Maryland; Chicago, Illinois; Forsyth County, North Carolina; Los Angeles County, California; Northern Manhattan, New York; and St. Paul, Minnesota). Details of the MESA cohort are published elsewhere.^63^ Briefly, 6814 adults of Chinese or Hispanic ethnicity or Black or White race who were between the ages of 45 and 84 years and free of clinical CVD were recruited for the study in 2000 to 2002 through population-based approaches. We used examination 1 (2000-2002) demographic, health, and tract information and examination 5 (2010-2011) chronological age and blood-based DNA methylation data to account for likely lag in the association between social exposures and epigenomic changes.

MESA staff purified monocytes from the blood samples of a random subset of 1264 patients of 4 MESA sites (Maryland, North Carolina, New York, and Minnesota) at examination 5. Details on sample processing and preprocessing of DNA methylation data are provided in the eMethods in Supplement 1.^64^ We excluded 100 participants with monocyte fractions less than 90%, as it reflects unwanted technical variation. DNA methylation age, in years, was calculated using 4 clock algorithms, 2 created to capture chronological age and 2 created to capture physiological dysregulation.^53^ To estimate chronological age, we used 353 DNA methylation sites following the Horvath,^54,65,66,67^ and 71 sites following the Hannum et al.^55^ We used 513 sites that comprised markers of tissue and immune function and chronological age for the PhenoAge clock, following Levine et al.^56^ Finally, we used 1030 sites that comprised markers related the function of numerous physiological systems and pack-years of smoking for the GrimAge clock, following Lu et al.^57^

### Dependent Variables

For each clock, DMAA was calculated as the residual of the regression of DNA methylation age on chronological age,^54^ since the raw difference between DNA methylation age and chronological age was associated with chronological age, while the residuals were not (eFigure 1 in Supplement 1). To examine bivariate associations between DNA methylation age and other variables, we dichotomized GrimAge DMAA, categorizing accelerating aging as a residual of 0 or more, indicating that the DNA methylation age was greater than chronological age. We selected this clock because the literature suggests it has a highly robust association with morbidity and mortality.^68^

### Independent Variables

Tract-level racial and ethnic segregation and poverty information from the 2000 census was linked to examination 1 tracts. To capture tract-level racial and ethnic residential segregation, we used the Getis-Ord *G* statistic (G_i_*).^69,70^ In MESA, G_i_* is the tract-level racial and ethnic composition, with a distance-decayed 1-mile radius buffer around its centroid within the Core Based Statistical Area, which is a Census Bureau–defined area containing at least 1 urbanized core along with socioeconomically integrated adjacent counties (eg, a metropolitan area). The census and MESA both include information on Black or White race and Hispanic ethnicity. We combined this information to create groups that reflect potential sociopolitical inequities. From the census data, we used information on Black race with or without Hispanic ethnicity (ie, Black); Hispanic ethnicity of any race (ie, Hispanic); and White race without Hispanic ethnicity (ie, non-Hispanic White). The G_i_* is a *z*-score, with greater values representing greater clustering segregation. G_i_* scores vary by race and ethnicity, with a range of −4.58 to 9.34 for non-Hispanic White individuals, −3.58 to 7.74 for non-Hispanic Black individuals, and −5.24 to 11.73 for Hispanic individuals. To create mutually exclusive groups in MESA while reflecting potential sociopolitical inequities, we used information on Black race without Hispanic ethnicity (ie, non-Hispanic Black), Hispanic of any race, and White race without Hispanic ethnicity. Following the literature, we matched the segregation measure to participant race and ethnicity. We modeled G_i_* and continuous and categorical variables based on normal distribution critical values corresponding to *P* < .05 and *P* < .01 (eMethods in Supplement 1). Tract-level poverty data were used to calculate the percentage of persons below the poverty level. For regression models, we standardized the poverty measure for comparability to the segregation measure.

### Statistical Analysis

Continuous variables were described using mean and SD; count variables, median and IQR; and categorical variables, number and percentages. Participants were excluded for missing information, yielding an analytic sample size of 1102 individuals (eFigure 2 in Supplement 1). We compared the excluded and analytic samples using *t* tests for continuous variables and χ^2^ tests for categorical variables (eTable 1 in Supplement 1). All subsequent analyses were reported stratified by baseline self-reported race and ethnicity due to little overlap in segregation values of non-Hispanic Black and non-Hispanic White participants. When tract poverty was the focal exposure, we estimated models with all racial and ethnic groups together for consistency with the extant literature and stratified by race and ethnicity to provide information for the models that included poverty in the interaction term.

Using ordinary least squares regression, we estimated models to evaluate the associations of segregation, poverty, or their interaction with DMAA stratified by race and ethnicity. In model 1, we estimated the unadjusted association between segregation or poverty and DMAA. We adjusted for the cell type and baseline self-reported gender (model 2) for precision. We adjusted for the potential confounders of maternal and own education (model 3), which were participant-reported at examination 1. We adjusted for study site, which might capture unmeasured confounders (model 4A), but removed it when adjusting for tract-level confounders (model 4B) of poverty or segregation (whichever was not the focal exposure), measured at examination 1. While study site may capture confounders between the tract-level exposure and outcomes, it may also be a driver of these exposures. For model 5, we used all the factors included in model 4B and included the potential mediators of smoking, alcohol use, body mass index, and a count of chronic conditions, all measured at examination 1. To account for multiple comparisons, we used the Benjamini-Hochberg correction to *P* values.^71^

To examine the modifying role of tract poverty on the association between segregation and DMAA, we fit a model from model 4B with a term for the poverty-segregation multiplicative interaction. All models use tract-based clustered standard errors.

We conducted 4 sensitivity analyses. First we estimated models with participants who were younger than 55 years at baseline (377 participants), since the MESA cohort was CVD-free at their baseline ages of 45 to 84 years and may have represented a particularly healthy group of adults at older ages. Second, we used models with participants who did not move between 2000 and 2010 (834 participants) to focus on those who may have had a more consistent neighborhood exposure over the follow-up period. Third, we created models using health and health behavior information from examination 1, rather than examination 5, in an attempt to adjust for factors that might be correlated with DNA methylation age clocks at baseline. Fourth, we used a categorical version of the segregation measures to reflect the statistical significance in clustering.

*P* values were 2-sided, and statistical significance was set at *P* < .05. Analyses were performed in R software version 4.1.0 (R Project for Statistical Computing). Data were analyzed from May 2021 to October 2023. Code for all analyses and figures are available elsewhere.^72^

## Results

A total of 1102 participants (mean [SD] age, 69.7 [9.4] years; 562 [51%] women) were included, with 348 Hispanic participants, 222 non-Hispanic Black participants, and 533 non-Hispanic White participants (Table 1). The 162 excluded participants and analytic samples differed in some measures: excluded participants were more likely to live in tracts with high poverty levels and included more non-Hispanic Black participants and fewer non-Hispanic White participants (eTable 1 in Supplement 1).

**Table 1.** Participant Characteristics by Race and Ethnicity

Characteristic^a^	Participants, No. (%)				*P* value^b^
	Overall (N = 1102)	Hispanic (n = 348)	Non-Hispanic Black (n = 221)	Non-Hispanic White (n = 533)	
GrimAge age acceleration, mean (SD), y	−0.02 (4.09)	−0.25 (3.73)	0.83 (4.51)	−0.22 (4.11)	.003
GrimAge direction					
Accelerated	551 (50)	134 (39)	111 (50)	219 (41)	.02
Decelerated	551 (50)	214 (61)	110 (50)	314 (59)	
Hannum age acceleration, mean (SD), y	0.00 (4.60)	−0.21 (4.39)	−1.65 (4.99)	0.83 (4.42)	<.001
Horvath age acceleration, y	0.00 (3.39)	−0.33 (3.13)	−0.10 (4.05)	0.26 (3.24)	.04
PhenoAge age acceleration, mean (SD), y	0.00 (5.81)	−0.07 (5.66)	−0.16 (6.06)	0.14 (5.81)	.77
Tract segregation G_i_*, mean (SD), *z*-score^c^	NC	5.11 (4.34)	2.53 (2.49)	−0.54 (2.40)	NC^d^
Tract poverty, percentage^e^	0.16 (0.12)	0.21 (0.13)	0.22 (0.14)	0.11 (0.07)	<.001
Chronologic age, mean (SD), y	69.7 (9.44)	68.6 (9.48)	70.3 (8.91)	70.2 (9.57)	.03
Gender					
Men	540 (49)	171 (49)	91 (41)	278 (52)	.03
Women	562 (51)	177 (51)	130 (59)	255 (48)	
Education, self					
≤High school	372 (34)	184 (53)	73 (33)	115 (22)	<.001
Some college	298 (27)	94 (27)	73 (33)	131 (25)	
≥College	432 (39)	70 (20)	75 (34)	287 (54)	
Education, mother					
<High school	584 (53)	271 (78)	116 (53)	197 (37)	<.001
High school	320 (29)	63 (18)	68 (31)	189 (35)	
>High school	198 (18)	14 (4)	37 (17)	147 (28)	
Smoking status					
Current	90 (8)	21 (6)	27 (12)	42 (8)	.07
Former	563 (51)	181 (52)	99 (45)	283 (53)	
Never	449 (41)	146 (42)	95 (43)	208 (39)	
Current alcohol use	694 (63)	190 (55)	121 (55)	383 (72)	<.001
BMI, mean (SD)	29.1 (5.09)	29.5 (4.72)	30.5 (5.47)	28.4 (5.04)	<.001
Chronic conditions, median (IQR), No.	1.0 (0.0-1.0)	1.0 (0.0-1.0)	1.0 (0.0-2.0)	1.0 (0.0-1.0)	<.001
Monocytes, mean (SD), %^f^	0.96 (0.02)	0.96 (0.02)	0.95 (0.02)	0.96 (0.02)	<.001

Abbreviations: BMI, body mass index (calculated as weight in kilograms divided by height in meters squared); G_i_*, Getis-Ord segregation index; NC, not calculated.

^a^ Chronological age, DNA methylation and leukocyte type proportion (monocytes, CD8+, B cells) were collected/measured at examination 5; all other information was collected at examination 1.

^b^ *P* value for difference among racial and ethnic groups.

^c^ G_i_* was calculated for Hispanic, non-Hispanic Black, and non-Hispanic White participants as the segregated clustering of Hispanic, non-Hispanic Black, and non-Hispanic White residents by tract, respectively.

^d^ Tests for significant difference in the segregation measures was not calculated across racial and ethnic groups because the segregation measures were different for each group.

^e^ Represents percentage of those in the tract living at or below the poverty level by tract of the individual participant’s tract.

^f^ Represents individual sample estimated monocyte proportions.

At examination 5, DMAA differed by clock and race and ethnicity. For the Horvath and Hannum clocks, Hispanic and non-Hispanic Black participants showed age deceleration, while non-Hispanic White participants showed age acceleration (Table 1). With the GrimAge clock, Hispanic and non-Hispanic White participants showed age deceleration while non-Hispanic Black participants showed age acceleration (Table 1).

At examination 1, Hispanic and non-Hispanic Black participants lived in tracts with greater clustering of residents of their own race and ethnicity than would be expected, given the racial compositions of their cities (Table 1). For example, the mean (SD) *z*-score G_i_* for non-Hispanic Black participants of 2.53 (2.49) indicated that in general, non-Hispanic Black participants lived in tracts with more than 2 SDs higher clustering of Black residents than would be expected by the overall percentage of Black residents in their respective cities. On the other hand, non-Hispanic White participants lived in tracts that did not have any clustering of non-Hispanic White residents. At examination 1, Hispanic and non-Hispanic Black participants lived in tracts where approximately 20% of the residents lived in poverty, while non-Hispanic White participants lived in tracts where 10% of residents lived in poverty. Segregation and poverty were associated in this sample, but this association varied by race and ethnicity. For non-Hispanic White participants, greater clustering of non-Hispanic White residents was associated with fewer residents living in poverty. For Hispanic and non-Hispanic Black participants, greater clustering of residents of their own race and ethnicity was associated with a greater percentage of residents living in poverty (eFigure 3 in Supplement 1).

Among non-Hispanic Black (but not Hispanic) participants, those who showed a DMAA lived in more highly segregated tracts than those who showed a decelerated age (mean [SD] G_i_*: acceleration group, 3.03 [2.59]; deceleration group, 2.03 [2.30]; *P* = .003) (Table 2). Similarly, among Hispanic participants, those who showed DMAA were more likely to live in tracts with higher levels of poverty compared with those who showed a decelerated age (mean [SD] tract poverty level: acceleration group, 0.24 [0.14]; deceleration group, 0.20 [0.13]; *P* = .02) (Table 2). For non-Hispanic White participants, there were no differences in either segregation or poverty levels in those who showed an accelerated vs decelerated age (mean [SD] G_i_*: acceleration group, −0.64 [2.30]; deceleration group, −0.47 [2.47]; *P* = .41; mean [SD] tract poverty level: acceleration group, 0.11 [0.07]; deceleration group, 0.11 [0.08]; *P* = .85).

**Table 2.** Participant Characteristics by Race and Ethnicity and GrimAge DNA Methylation Age Group

Measure^a^	Participants, No. (%)								
	Hispanic			Non-Hispanic Black			Non-Hispanic White		
	DMAA (n = 134)	DMAD (n = 214)	*P* value^b^	DMAA (n = 111)	DMAD (n = 110)	*P* value^b^	DMAA (n = 219)	DMAD (n = 314)	*P* value^b^
Hannum age acceleration, mean (SD), y	0.86 (4.93)	−0.88 (3.87)	.001	−0.95 (5.37)	−2.35 (4.50)	.04	1.71 (4.87)	0.21 (3.96)	<.001
Horvath age acceleration, mean (SD), y	0.40 (3.41)	−0.79 (2.85)	.001	0.21 (4.29)	−0.42 (3.78)	.25	0.62 (3.69)	0.01 (2.86)	.04
PhenoAge age acceleration, mean (SD), y	1.73 (5.60)	−1.20 (5.42)	<.001	0.32 (6.22)	−0.64 (5.89)	.24	2.03 (5.85)	−1.18 (5.41)	<.001
Tract segregation G_i_*, mean (SD), *z*-score^c^	4.87 (4.25)	5.26 (4.39)	.41	3.03 (2.59)	2.03 (2.30)	.003	−0.64 (2.30)	−0.47 (2.47)	.41
Tract poverty^d^	0.24 (0.14)	0.20 (0.13)	.02	0.24 (0.15)	0.20 (0.14)	.10	0.11 (0.07)	0.11 (0.08)	.85
Age, mean (SD), y	69.9 (9.77)	67.8 (9.21)	.04	69.9 (8.62)	70.7 (9.21)	.53	69.9 (9.07)	70.4 (9.92)	.54
Gender									
Men	95 (71)	75 (35)	<.001	60 (54)	30 (27)	<.001	153 (70)	122 (39)	<.001
Women	39 (29)	139 (65)		51 (46)	80 (73)		66 (30)	192 (61)	
Education, self									
≤High school	70 (52)	113 (53)	.60	33 (30)	39 (36)	.66	57 (26)	60 (19)	.001
>High school	34 (25)	62 (29)		38 (34)	35 (32)		66 (30)	66 (21)	
≥College	30 (22)	39 (18)		40 (36)	36 (33)		96 (44)	188 (60)	
Education, mother									
<High school	101 (75)	169 (79)	.63	57 (51)	59 (54)	.68	85 (39)	110 (35)	.34
High school	28 (21)	36 (17)		33 (30)	35 (32)		79 (36)	110 (35)	
>High school	5 (4)	9 (4)		21 (19)	16 (15)		55 (25)	94 (30)	
Smoking status									
Current	17 (13)	4 (2)	<.001	23	2	<.001	42 (19)	3 (1)	<.001
Former	81 (60)	101 (47)		25 (23)	2 (2)		129 (59)	151 (48)	
Never	36 (27)	109 (51)		57 (51)	43 (39)		48 (22)	160 (51)	
Never drank alcohol	17 (13)	60 (28)	.003	29 (26)	65 (59)	<.001	11 (5)	28 (9)	.10
BMI, mean (SD)	29.1 (4.18)	29.7 (5.03)	.29	30.4 (5.15)	30.6 (5.80)	.78	29.1 (5.05)	27.9 (4.99)	.01
Chronic conditions, median (IQR), No.^e^	1.0 (0.0 to 1.8)	1.0 (0.0 to 1.0)	.40	1.0 (0.0 to 2.0)	1.0 (0.0 to 2.0)	.30	1.0 (0.0 to 1.0)	1.0 (0.0 to 1.0)	.56
Monocytes, mean (SD), %	0.96 (0.02)	0.96 (0.02)	.03	0.95 (0.02)	0.95 (0.02)	.16	0.96 (0.02)	0.96 (0.02)	.06

Abbreviations: BMI, body mass index (calculated as weight in kilograms divided by height in meters squared); DMAA, DNA methylation age acceleration; DMAD, DNA methylation age deceleration; G_i_*, Getis-Ord segregation index.

^a^ Chronological age, DNA methylation, and leukocyte type proportion (monocytes, CD8+, B cells) were collected at examination 5; all other information was collected at examination 1.

^b^ *P* value for difference between DMAA and DMAD groups.

^c^ G_i_* was calculated for Hispanic, non-Hispanic Black, and non-Hispanic White participants as the segregated clustering of Hispanic, Black, and non-Hispanic White residents by tract, respectively.

^d^ Represents percentage of those in the tract living at or below the poverty level by tract of the individual participant’s tract.

^e^ Represents the sum of the following chronic conditions: arthritis, cancer, diabetes, hepatitis, hypertension, and kidney disease.

Segregation was associated with later DMAA using the GrimAge and PhenoAge clocks, but not the Hannum and Horvath clocks, and only for non-Hispanic Black participants. For example, for each 1-SD greater segregation, there was a nearly half-year greater DMAA for the GrimAge clock (β = 0.42 [95% CI, 0.20-0.64] years; adjusted *P* < .001) (Table 3). While attenuated, this association remained after adjustment for cell type proportion, gender, maternal and own education, and tract poverty (β = 0.39 [95% CI, 0.20-0.58] years; adjusted *P* = .003). After adjustment for health and health behaviors, either at examination 1 or 5, this association was further attenuated (Table 3). Clustering of either Hispanic or non-Hispanic White residents was not associated with any DMAA clock for Hispanic or non-Hispanic White participants (Table 3).

**Table 3.** Association Between Tract Racial and Ethnic Segregation and DNA Methylation Age Acceleration by Race and Ethnicity

Model^a^	Hispanic			Non-Hispanic Black			Non-Hispanic White		
	β (95%CI), y	*P* value^b^	β (95%CI), y		*P* value^b^	β (95%CI), y		*P* value^b^
**GrimAge DNA methylation age acceleration**										
1	−0.07 (−0.15 to 0.02)	.22	0.42 (0.20 to 0.64)		<.001	−0.12 (−0.26 to 0.02)		.22
2	−0.01 (−0.09 to 0.07)	.89	0.37 (0.20 to 0.55)		<.001	−0.12 (−0.28 to 0.08)		.28
3	−0.01 (−0.09 to 0.07)	.86	0.39 (0.20 to 0.58)		.003	−0.08 (−0.24 to 0.07)		.48
4A	−0.01 (−0.09 to 0.07)	.97	0.39 (0.19 to 0.59)		.001	−0.09 (−0.25 to 0.08)		.62
4B	−0.06 (−0.16 to 0.04)	.39	0.30 (0.10 to 0.50)		.02	−0.02 (−0.20 to 0.16)		.86
5	−0.07 (−0.17 to 0.04)	.40	0.20 (0.01 to 0.39)		.12	−0.02 (−0.18 to 0.13)		.82
**Hannum DNA methylation age acceleration**										
1	−0.08 (−0.21 to 0.05)	.35	−0.04 (−0.23 to 0.16)		.78	−0.07 (−0.22 to 0.08)		0.50
2	−0.04 (−0.16 to 0.09)	.72	−0.05 (−0.25 to 0.15)		.72	−0.08 (−0.23 to 0.07)		0.45
3	−0.04 (−0.17 to 0.09)	.77	−0.12 (−0.34 to 0.10)		.44	−0.06 (−0.21 to 0.09)		0.66
4A	−0.02 (−0.11 to 0.08)	.96	−0.12 (−0.34 to 0.10)		.62	−0.03 (−0.21 to 0.15)		0.96
4B	0.06 (−0.06 to 0.17)	.45	−0.05 (−0.29 to 0.18)		.74	−0.07 (−0.27 to 0.12)		0.56
5	0.06 (−0.06 to 0.17)	.49	−0.05 (−0.29 to 0.18)		.75	−0.07 (−0.27 to 0.13)		0.57
**Horvath DNA methylation age acceleration**										
1	−0.03 (−0.11 to 0.04)	.53	−0.14 (−0.33 to 0.05)		.28	−0.07 (−0.18 to 0.05)		.40
2	−0.02 (−0.10 to 0.06)	.72	−0.13 (−0.32 to 0.06)		.32	−0.06 (−0.18 to 0.06)		.51
3	−0.02 (−0.09 to 0.06)	.83	−0.15 (−0.35 to 0.05)		.26	−0.06 (−0.18 to 0.07)		.56
4A	−0.01 (−0.08 to 0.06)	.96	−0.14 (−0.36 to 0.08)		.50	0.01 (−0.12 to 0.14)		.97
4B	0.04 (−0.03 to 0.11)	.39	−0.03 (−0.23 to 0.16)		.80	−0.09 (−0.24 to 0.06)		.39
5	0.05 (−0.02 to 0.12)	.30	−0.01 (−0.23 to 0.21)		.90	−0.08 (−0.22 to 0.07)		.47
**PhenoAge DNA methylation age acceleration**										
1	−0.04 (−0.18 to 0.10)	.62	0.29 (0.02 to 0.57)		.14	−0.07 (−0.25 to 0.11)		.56
2	−0.04 (−0.18 to 0.11)	.72	0.30 (0.01 to 0.59)		.13	−0.07 (−0.26 to 0.12)		.69
3	−0.02 (−0.17 to 0.12)	.83	0.27 (−0.04 to 0.58)		.23	−0.04 (−0.23 to 0.15)		.83
4A	0.00 (−0.15 to 0.14)	.97	0.27 (−0.04 to 0.58)		.28	0.04 (−0.20 to 0.27)		.96
4B	0.04 (−0.11 to 0.20)	.68	0.35 (0.00 to 0.70)		.14	−0.10 (−0.34 to 0.14)		.44
5	0.07 (−0.09 to 0.23)	.50	0.35 (0.02 to 0.68)		.12	−0.09 (−0.33 to 0.14)		.52

^a^ Model 1 was estimated with no covariates; model 2, leukocyte type proportion (monocytes, CD8+, B cells) and gender; model 3, model 2 covariates plus self education level and maternal education level; model 4A, model 3 covariates plus site; model 4B, model 3 covariates plus tract poverty; model 5, model 4B covariates plus baseline information on smoking status, never drank alcohol, body mass index, and count of chronic conditions.

^b^ *P* values are adjusted for multiple tests using the Benjamini-Hochberg correction.

The association between tract poverty at baseline and DMAA at examination 5 in the whole sample showed divergent results based on the clock used (Table 4). Using the clocks that were trained to capture physiological dysregulation, there was a positive association between tract poverty and GrimAge but not PhenoAge DMAA. However, there was an inverse association when using Hannum and Horvath clocks. We found no association when using the PhenoAge clock.

**Table 4.** Association Between Tract Poverty and DNA Methylation Age Acceleration by Race and Ethnicity

Model^a^	Total sample		Hispanic		Non-Hispanic Black		Non-Hispanic White	
	β (95%CI), y	*P* value^b^	β (95%CI), y	*P* value^b^	β (95%CI), y	*P* value^b^	β (95%CI), y	*P* value^b^
**GrimAge DNA methylation age acceleration**								
1	0.45 (0.20 to 0.71)	.005	0.20 (−0.15 to 0.55)	.40	0.73 (0.20 to 1.26)	.03	0.47 (−0.08 to 1.02)	.22
2	0.51 (0.28 to 0.75)	<.001	0.27 (−0.04 to 0.58)	.22	0.79 (0.32 to 1.26)	.005	0.48 (−0.09 to 1.05)	.22
3	0.46 (0.21 to 0.72)	.004	0.28 (−0.03 to 0.59)	.23	0.75 (0.28 to 1.22)	.01	0.48 (−0.03 to 0.99)	.18
4A	0.66 (0.39 to 0.93)	<.001	0.62 (0.13 to 1.11)	.10	0.99 (0.52 to 1.46)	.001	0.49 (0.02 to 0.96)	.16
4B	0.48 (0.23 to 0.74)	.006	0.40 (0.01 to 0.79)	.14	0.48 (−0.01 to 0.97)	.14	0.45 (−0.10 to 1.00)	.24
5	0.47 (0.24 to 0.71)	.003	0.41 (0.02 to 0.80)	.12	0.61 (0.18 to 1.04)	.03	0.28 (−0.27 to 0.83)	.47
**Hannum DNA methylation age acceleration**								
1	−0.66 (−0.93 to −0.39)	<.001	−0.61 (−1.02 to −0.20)	.02	−0.38 (−0.91 to 0.15)	.28	0.09 (−0.44 to 0.62)	.78
2	−0.59 (−0.86 to −0.32)	<.001	−0.58 (−0.99 to −0.17)	.03	−0.29 (−0.82 to 0.24)	.49	0.10 (−0.41 to 0.61)	.78
3	−0.41 (−0.70 to −0.12)	.02	−0.61 (−1.00 to −0.22)	.01	−0.44 (−0.99 to 0.11)	.26	0.08 (−0.43 to 0.59)	.83
4A	−0.16 (−0.47 to 0.15)	.62	0.22 (−0.27 to 0.71)	.71	−0.43 (−1.00 to 0.14)	.44	0.15 (−0.40 to 0.70)	.94
4B	−0.38 (−0.67 to −0.09)	.04	−0.73 (−1.12 to −0.34)	.006	−0.39 (−0.98 to 0.20)	.39	−0.09 (−0.73 to 0.56)	.82
5	−0.36 (−0.65 to −0.07)	.07	−0.69 (−1.12 to −0.26)	.01	−0.38 (−1.01 to 0.57)	.43	−0.06 (−0.71 to 0.59)	.89
**Horvath DNA methylation age acceleration**								
1	−0.42 (−0.61 to −0.23)	<.001	−0.35 (−0.66 to −0.04)	.10	−0.67 (−1.08 to −0.26)	.008	−0.04 (−0.45 to 0.37)	.85
2	−0.40 (−0.60 to −0.20)	<.001	−0.38 (−0.69 to −0.07)	.07	−0.61 (−1.04 to −0.18)	.03	−0.03 (−0.42 to 0.36)	.89
3	−0.40 (−0.62 to −0.18)	.004	−0.37 (−0.68 to −0.06)	.06	−0.68 (−1.13 to −0.23)	.02	−0.03 (−0.43 to 0.36)	.87
4A	−0.26 (−0.50 to −0.03)	.15	−0.18 (−0.61 to 0.25)	.72	−0.58 (−1.09 to −0.07)	.15	−0.09 (−0.46 to 0.29)	.94
4B	−0.38 (−0.60 to −0.16)	.006	−0.46 (−0.75 to −0.17)	.02	−0.65 (−1.12 to −0.18)	.04	−0.24 (−0.73 to 0.25)	.45
5	−0.38 (−0.60 to −0.16)	.009	−0.52 (−0.83 to −0.21)	.009	−0.70 (−1.19 to −0.21)	.03	−0.21 (−0.70 to 0.28)	.50
**PhenoAge DNA methylation age acceleration**								
1	−0.31 (−0.62 to 0.00)	.15	−0.43 (−0.96 to 0.10)	.22	−0.21 (−0.84 to 0.42)	.60	−0.23 (−0.86 to 0.40)	.57
2	−0.27 (−0.60 to 0.06)	.22	−0.48 (−1.03 to 0.07)	.22	−0.09 (−0.74 to 0.55)	.84	−0.19 (−0.84 to 0.46)	.72
3	−0.30 (−0.67 to 0.07)	.26	−0.44 (−1.03 to 0.15)	.26	−0.13 (−0.80 to 0.54)	.83	−0.20 (−0.83 to 0.43)	.74
4A	−0.01 (−0.42 to 0.40)	.97	0.41 (−0.39 to 1.21)	.62	−0.01 (−0.72 to 0.63)	.97	−0.17 (−0.84 to 0.50)	.94
4B	−0.31 (−0.68 to 0.06)	.23	−0.53 (−1.18 to 0.12)	.23	−0.43 (−1.16 to 0.30)	.39	−0.43 (−1.21 to 0.35)	.41
5	−0.30 (−0.67 to 0.07)	.23	−0.68 (−1.31 to −0.05)	.12	−0.42 (−1.13 to 0.29)	.44	−0.38 (−1.16 to 0.40)	.49

^a^ Model 1 was estimated with no covariates; model 2, leukocyte type proportion (monocytes, CD8+, B cells) and gender; model 3, model 2 covariates plus self education level and maternal education level; model 4A, model 3 covariates plus site; model 4B, model 3 covariates plus racial and ethnic segregation; model 5, model 4B covariates plus baseline information on smoking status, never drank alcohol, body mass index, and count of chronic conditions.

^b^ *P* values are adjusted for multiple tests using the Benjamini-Hochberg correction.

There was a positive association between tract poverty and GrimAge DMAA across all racial and ethnic groups, and this was particularly consistent for non-Hispanic Black participants. Among non-Hispanic Black participants, for each 1-SD greater poverty, there was an approximately three-quarters year greater DMAA after adjustment for leukocyte type proportion, gender, maternal and own education, and segregation, (β = 0.75 [95% CI, 0.28-1.22] years; adjusted *P* = .01) (Table 4).

For Hispanic and non-Hispanic Black participants, but not non-Hispanic White participants, there was generally an inverse association between tract poverty and DMAA when using the Hannum or Horvath clocks (Table 4). These associations generally remained consistent after adjustment for covariates.

The association between segregation and DMAA in GrimAge was modified by tract poverty for Hispanic and non-Hispanic Black participants but not non-Hispanic White participants. For non-Hispanic Black participants, greater segregation of Black residents was associated with greater DMAA for those living in tracts with higher levels of poverty compared with lower levels (interaction term, 0.24; 95% CI, 0.07-0.42; *P* = .006) (Figure; eTable 2 in Supplement 1). On the other hand, for Hispanic participants, the association between segregation of Hispanic residents and DMAA was the inverse for Hispanic participants living in high poverty tracts vs low poverty tracts (Figure; eTable 2 in Supplement 1). In the overall sample, census tract poverty level was associated with GrimAge DNA methylation age acceleration (β = 0.45; 95% CI, 0.20-0.71; adjusted *P* = .005). The association between segregation and DMAA using the other clocks was not modified by poverty (Figure; eTable 2 and eFigure 4 in Supplement 1).

**Figure.**
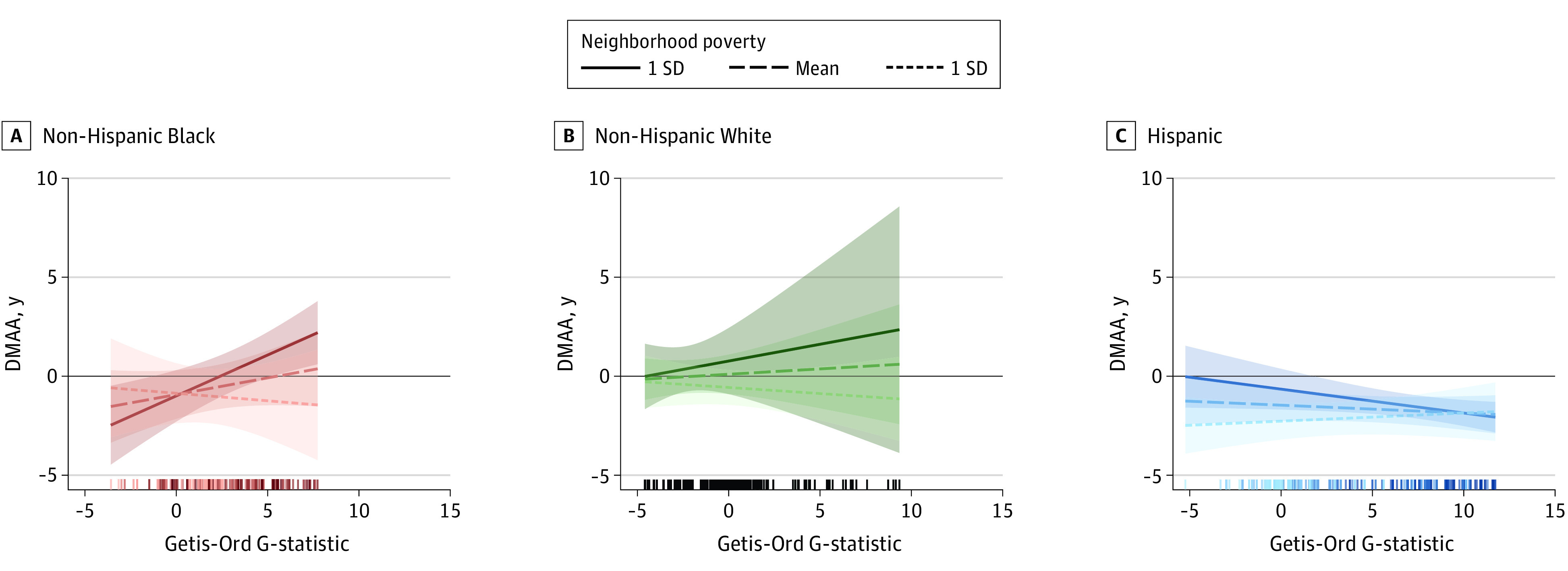
GrimAge DNA Methylation Age Acceleration (DMAA) as a Function of Residential Racial Segregation by Race and Ethnicity and Tract Poverty

When we included health and behavior information from examination 1 rather than examination 5, our results were nearly identical in the coefficients, 95% CIs, and adjusted *P* values (eTable 3 and eTable 4 in Supplement 1). In models with only participants younger than 55 years at examination 1, the pattern of associations was similar to that in the entire MESA sample, but the standard errors were larger (eTable 3 in Supplement 1). In models with only participants who did not move between 2000 and 2010, the coefficients were nearly identical to those in the entire MESA sample, with smaller standard errors and adjusted *P* values than for the entire MESA sample (eTable 6 and eTable 7 in Supplement 1). Using the categorical segregation measures corresponding to statistically significant clustering yielded qualitatively similar results, particularly for non-Hispanic Black participants (eTable 5 in Supplement 1).

## Discussion

This cohort study examined the associations of neighborhood segregation and poverty with later DMAA. The pattern of results varied by participant and neighborhood race and ethnicity and by DMAA clock. For non-Hispanic Black participants, higher levels of segregation of non-Hispanic Black residents was associated with GrimAge DMAA. This was particularly pronounced for participants who lived in very segregated tracts. Neighborhood segregation of Black residents has been associated with cardiovascular risk, including blood pressure, incident CVD, and a cardiometabolic risk index.^26,27,73^ Both the PhenoAge and GrimAge (in particular) clocks estimate multiple morbidities and mortality.^68^

Our results suggest that this association of segregation with DMAA was pronounced for poorer tracts. It has been well documented that the segregation of Black individuals in the US is compounded by poverty to result in highly underresourced neighborhoods with particularly poor health outcomes.^62^ The literature suggests that even middle-class Black individuals are segregated into neighborhoods with fewer resources compared with neighborhoods with poorer White residents.^60,74,75^ Middle-class Black individuals may have to navigate spaces of predominantly White individuals in their schools, stores, and workplaces. Thus, despite the fewer neighborhood resources, there may be psychosocial benefits for middle class Black individuals to live among Black neighbors, such as lower hypervigilance used when navigating spaces of predominantly White individuals.^10,76,77^

Some researchers have examined the association between other neighborhood measures and DNA methylation patterns. In a sample of 157 residents of Detroit, Michigan (88% Black adults; 11% White adults; mean age, 53.3 years), neighborhood poverty was not associated with PhenoAge DMAA (but GrimAge was not assessed).^58^ However, an index created from neighborhood features, such as the presence of abandoned cars and graffiti, was associated with PhenoAge DMAA.^58,78^ Because the PhenoAge clock is not consistently associated with social factors, even in the same sample, it would be interesting to examine whether the GrimAge clock is associated with neighborhood poverty (or neighborhood segregation) in this sample.

While Hispanic participants were more likely to live in segregated neighborhoods, neighborhood segregation was not associated with either GrimAge or PhenoAge DMAA. The association between Hispanic segregation and health is inconsistent^79^ and likely due to differences in the social, economic, and political meaning of Hispanic or Latino segregation by ethnicity (eg, Mexican, Puerto Rican, or Argentine background) across time and place. Higher vs lower neighborhood poverty was associated with GrimAge DMAA in Hispanic participants; however, our results further suggest that for individuals living in poor neighborhoods, segregation was associated with GrimAge DNA methylation age deceleration. This is consistent with reports that greater density of Hispanic residents is associated with better health for those living in poor neighborhoods.^80^ However, this interactive association between segregation and poverty is not robust across studies,^81,82^ further suggesting the importance of Hispanic or Latino group meaning.

That neighborhood poverty was not associated with DMAA for non-Hispanic White participants was unexpected, as neighborhood poverty has been associated with numerous poor health outcomes for White individuals in the US. In a sample of 2630 non-Hispanic White women (mean age, 57.8 years), researchers reported that living in disadvantaged neighborhoods was associated with greater GrimAge and PhenoAge DMAA.^59^ Notably, this association was not linear; residence in neighborhoods of any quartile above the lowest disadvantage quartile was associated with greater DMAA.^59^ We did not find any significant association, but there may be a nonlinear association, as this other study would suggest.

When using the clocks that were trained to capture chronological age (ie, Hannum, Horvath), living in neighborhoods with higher levels of poverty was associated with a DNA methylation age deceleration compared with living in neighborhoods with lower poverty for Hispanic and non-Hispanic Black participants. While studies using Hannum and Horvath clocks have yielded mixed results, none have shown this inverse association. In the sample of non-Hispanic White women assessed by Lawrence et al,^59^ compared with living in neighborhoods characterized by the highest quartile of disadvantage, living in other neighborhoods was associated with Hannum but not Horvath DMAA. Furthermore, in a sample of 99 Black women (mean age, 48.5 years), a neighborhood disadvantage index was associated with Hannum DMAA even when adjusting for potential neighborhood selection bias, using marginal structural models.^48^ Yet, in the Detroit, Michigan, sample assessed by Martin et al,^58^ neighborhood poverty was not associated with Hannum or Horvath DMAA. The inverse association shown in our chronologic age clock results may be due to the particular characteristics of the MESA sample, who were CVD-free at baseline. Neighborhood socioeconomic status is inversely associated with incident CVD,^83^ CVD mortality,^84^ and CVD risk factors.^85,86,87^ It may be that MESA participants who live in high-poverty neighborhoods but had not yet shown signs of CVD particularly at older ages were a select group with different health trajectories.^88^

### Limitations

This study has some limitations. Foremost, these associations are cross-sectional. While we did not have epigenomic information at examination 1, we did have health and behavior information at examination 1, which might be associated with DNA methylation. If the clocks are tightly correlated with these measures of health and behaviors, then our results suggest that neighborhood poverty is associated with later GrimAge DMAA, even after adjustment for measures at examination 1. However, we use caution, as it requires the assumption that these clocks are tightly correlated with contemporaneous measures of health and behaviors.

Length of neighborhood residence may impact DNA methylation patterns. While we could not examine length of residence, our results are consistent for individuals who remained in the same neighborhood for the follow-up period.

This study had a large age range at baseline, and the association between neighborhood characteristics and DMAA may vary by baseline age. Furthermore, because MESA participants were CVD-free at baseline, they may have represented a relatively healthy sample, making our estimates conservative. We estimated models with only participants who were younger than 55 years at baseline, which showed a similar pattern of results; however future work could include a focus on the different age cohorts and samples that did not exclude those with CVD.

## Conclusions

The results of this cohort study suggest that for non-Hispanic Black participants, compared with living in other neighborhoods, living in neighborhoods characterized by greater segregation of Black residents or by greater poverty was associated with accelerated epigenomic aging. Residential segregation has been an effective process by which different racial and ethnic groups have been sorted into neighborhoods of vastly unequal quality, with important implications for health. Clarifying the role of neighborhoods and identifying the specific features related to health are critical, as these features are neither random nor naturally occurring: they are amenable to policies and interventions^89^ and may be an effective strategy for promoting population health.^90^

## Notes

Supplement 1.
eMethods.
**eTable 1.** Participant Characteristics of Full, Analytic, and Excluded Samples, Multi-Ethnic Study of Atherosclerosis (2000-2010) **eTable 2.** Interactive Association Among Tract Racial Segregation, Tract Poverty, and DNA Methylation Age Acceleration by Race and Ethnicity, Multi-Ethnic Study of Atherosclerosis (2000-2010) **eTable 3.** Association Between Racial Segregation and DNA Methylation Age Acceleration by Race and Ethnicity, Multi-Ethnic Study of Atherosclerosis (2000-2010) Limited to Those With Baseline Age <55 **eTable 4.** Association Between Tract Poverty and DNA Methylation Age Acceleration by Race and Ethnicity, Multi-Ethnic Study of Atherosclerosis (2000-2010) Limited to Those With Baseline Age <55 **eTable 5.** Association Between Racial Segregation Categories and DNA Methylation Age Acceleration by Race and Ethnicity, Multi-Ethnic Study of Atherosclerosis (2000-2010) **eTable 6.** Association Between Racial Segregation and DNA Methylation Age Acceleration by Race/Ethnicity, Multi-Ethnic Study of Atherosclerosis (2000-2010) **eTable 7.** Association Between Tract Poverty and DNA Methylation Age Acceleration by Race/Ethnicity, Multi-Ethnic Study of Atherosclerosis (2000-2010) **eFigure 1.** GrimAge DNA Methylation Age Acceleration as Raw and Residual Values as a Function of Chronological Age, in Years, Multi-Ethnic Study of Atherosclerosis (2010) **eFigure 2.** Flow Chart of Inclusion Into Analytic Sample **eFigure 3.** Racial Segregation and Tract Poverty, by Race and Ethnicity, Multi-Ethnic Study of Atherosclerosis (2000-2010) **eFigure 4.** Hannum, Horvath, and PhenoAge DNA Methylation Age Acceleration as a Function of Racial Segregation by Race and Ethnicity and Tract Poverty, Multi-Ethnic Study of Atherosclerosis (2000-2010)

Supplement 2.
Data Sharing Statement
