# Supplementary material for: Racial and Ethnic Residential Segregation and Monocyte DNA Methylation Age Acceleration
Source: JAMA Netw Open. 2023 Nov 29;6(11):e2344722. doi: 10.1001/jamanetworkopen.2023.44722 (PMC10687663; doi:10.1001/jamanetworkopen.2023.44722)
Supplement: Supplement 1. — eMethods. eTable 1. Participant Characteristics of Full, Analytic, and Excluded Samples, Multi-Ethnic Study of Atherosclerosis (2000-2010) eTable 2. Interactive Association Among Tract Racial Segregation, Tract Poverty, and DNA Methylation Age Acceleration by Race and Ethnicity, Multi-Ethnic Study of Atherosclerosis (2000-2010) eTable 3. Association Between Racial Segregation and DNA Methylation Age Acceleration by Race and Ethnicity, Multi-Ethnic Study of Atherosclerosis (2000-2010) Limited to Those With Baseline Age <55 eTable 4. Association Between Tract Poverty and DNA Methylation Age Acceleration by Race and Ethnicity, Multi-Ethnic Study of Atherosclerosis (2000-2010) Limited to Those With Baseline Age <55 eTable 5. Association Between Racial Segregation Categories and DNA Methylation Age Acceleration by Race and Ethnicity, Multi-Ethnic Study of Atherosclerosis (2000-2010) eTable 6. Association Between Racial Segregation and DNA Methylation Age Acceleration by Race/Ethnicity, Multi-Ethnic Study of Atherosclerosis (2000-2010) eTable 7. Association Between Tract Poverty and DNA Methylation Age Acceleration by Race/Ethnicity, Multi-Ethnic Study of Atherosclerosis (2000-2010) eFigure 1. GrimAge DNA Methylation Age Acceleration as Raw and Residual Values as a Function of Chronological Age, in Years, Multi-Ethnic Study of Atherosclerosis (2010) eFigure 2. Flow Chart of Inclusion Into Analytic Sample eFigure 3. Racial Segregation and Tract Poverty, by Race and Ethnicity, Multi-Ethnic Study of Atherosclerosis (2000-2010) eFigure 4. Hannum, Horvath, and PhenoAge DNA Methylation Age Acceleration as a Function of Racial Segregation by Race and Ethnicity and Tract Poverty, Multi-Ethnic Study of Atherosclerosis (2000-2010) [file jamanetwopen-e2344722-s001.pdf]

Supplemental Online Content

Hicken MT, Dou J, Kershaw KN, Liu Y, Hajat A, Bakulski KM. Racial and ethnic residential segregation and monocyte DNA methylation age acceleration. *JAMA Netw Open*. 2023;6(11):e2344722. doi:10.1001/jamanetworkopen.2023.44722

eMethods.

eTable 1. Participant Characteristics of Full, Analytic, and Excluded Samples, Multi-Ethnic Study of Atherosclerosis (2000-2010)

eTable 2. Interactive Association Among Tract Racial Segregation, Tract Poverty, and DNA Methylation Age Acceleration by Race and Ethnicity, Multi-Ethnic Study of Atherosclerosis (2000-2010)

eTable 3. Association Between Racial Segregation and DNA Methylation Age Acceleration by Race and Ethnicity, Multi-Ethnic Study of Atherosclerosis (2000-2010) Limited to Those With Baseline Age <55

eTable 4. Association Between Tract Poverty and DNA Methylation Age Acceleration by Race and Ethnicity, Multi-Ethnic Study of Atherosclerosis (2000-2010) Limited to Those With Baseline Age <55

eTable 5. Association Between Racial Segregation Categories and DNA Methylation Age Acceleration by Race and Ethnicity, Multi-Ethnic Study of Atherosclerosis (2000-2010)

eTable 6. Association Between Racial Segregation and DNA Methylation Age Acceleration by Race/Ethnicity, Multi-Ethnic Study of Atherosclerosis (2000-2010)

eTable 7. Association Between Tract Poverty and DNA Methylation Age Acceleration by Race/Ethnicity, Multi-Ethnic Study of Atherosclerosis (2000-2010)

eFigure 1. GrimAge DNA Methylation Age Acceleration as Raw and Residual Values as a Function of Chronological Age, in Years, Multi-Ethnic Study of Atherosclerosis (2010)

eFigure 2. Flow Chart of Inclusion Into Analytic Sample

eFigure 3. Racial Segregation and Tract Poverty, by Race and Ethnicity, Multi-Ethnic Study of Atherosclerosis (2000-2010)

eFigure 4. Hannum, Horvath, and PhenoAge DNA Methylation Age Acceleration as a Function of Racial Segregation by Race and Ethnicity and Tract Poverty, Multi-Ethnic Study of Atherosclerosis (2000-2010)

This supplemental material has been provided by the authors to give readers additional information about their work.

© 2023 Hicken MT et al. *JAMA Network Open.*

eMethods

Full methodological description

This report follows the STROBE reporting guidelines for cross-sectional observational studies.

Dataset: The Multi-Ethnic Study of Atherosclerosis (MESA) is a prospective cohort in the US (Baltimore, MD; Chicago, IL; Forsyth County, NC; Los Angeles County, CA; Northern Manhattan, NY; and St. Paul, MN). Details of the MESA cohort are published elsewhere.[63] Briefly, 6,814 adults of White or Black race or Hispanic or Chinese ethnicity who were between the ages of 45-84 and free of clinical cardiovascular disease were recruited for the study in 2000-02 through population-based approaches. The current paper uses demographic, health, and tract (as a proxy for neighborhood) information collected at Exam 1 (2000-02) and chronological age and blood-based DNA methylation data collected at Exam 5 (2010-11). We selected an exposure at Exam 1 as there is likely a lag in the link between complex social exposures and epigenomic changes. Written informed consent at the time of participation and this secondary data analysis was approved by the University of Michigan Institutional Review Board (HUM00214985). MESA data are available by request (https://www.mesa-nhlbi.org/Publications.aspx).

DNA methylation preprocessing and variables: MESA staff purified monocytes from the blood samples of a random subset (n=1264) of four MESA sites (MN, NC, NY, and MN) at Exam 5. Sample processing and pre-processing of DNA methylation and gene expression data has been previously described.[64] Briefly, MESA staff isolated monocytes with magnetic beads from peripheral blood samples and assessed DNA methylation with the Illumina HumanMethylation450 BeadChip.[65] MESA then applied the following preprocessing procedures using the lumi package pipeline:[66] DNA methylation data were adjusted for red-green color bias and background correction was applied.[67] The DNA methylation data was quantile normalized. Probes with detection p-values>0.05 in >10% of samples were dropped (number of sites=630).

Preprocessed DNA methylation values were computed as M-values (the log ratio of methylated to unmethylated intensities) and provided to the manuscript authors. We converted the M-values to beta-values (an estimate of percent methylation), for biologic interpretability and compatibility with many downstream applications. Using the ewastools package[68] and an adult blood cell type reference panel,[69] we estimated proportions of six immune cell types: monocytes, B cells, CD4+ cells, CD8+ cells, granulocytes, and natural killer cells.[70] DNA methylation measures are impacted by sample cell type composition.[71] However, in MESA, cell composition may reflect unwanted technical variation in the monocyte cell enrichment laboratory process, thus we excluded participants whose samples contained an estimated monocyte fraction below 90% (n=100).

DNA methylation age, in years, was calculated using four different clock algorithms.[53] We used two clocks created to capture chronological age. First, following Horvath and colleagues[54,72-74] we used 353 DNA methylation sites, and adjusted for a broader array of cell type proportions when estimating chronological age. Second, following Hannum and colleagues[55] we used 71 sites to estimate chronological age. We used two clocks created to capture physiological dysfunction. Following Levine and colleagues, we used 513 sites that comprised markers of tissue and immune function and chronological age for the PhenoAge clock.[56] Finally, following Lu and colleagues, we used 1030 sites that comprised markers related the function of numerous physiological systems and pack-years of smoking for the GrimAge clock (https://dnamage.genetics.ucla.edu/home).[57]

Dependent variables: DNA methylation age acceleration ('DMAA') for each clock was calculated as the residual of the regression of DNA methylation age on chronological age.[54] We used the residuals as our primary outcome measure because the raw difference between DNA methylation age and chronological age was associated with chronological age, while the residuals were not (Supplemental Figure 1).

To examine bivariate associations between DNA methylation age and the exposure variables and covariates, we dichotomized DMAA using the GrimAge clock, categorizing accelerating aging as a residual ≥0, indicating that the DNA methylation age was equal to or greater than the chronological age. We selected this clock for these descriptive examinations as the literature suggests a highly robust association between this clock and morbidity and mortality.[75]

© 2023 Hicken MT et al. *JAMA Network Open.*

Independent variables: We used two exposure measures, at Exam 1, that capture different aspects of the neighborhood sociodemographic composition, racial segregation and poverty at the tract level from the 2000 census. While many use racial composition (e.g., percent Black race) as a proxy for tract racial residential segregation, it does not account for the overall racial composition of the city.[76] Further, it does not account for the spatial clustering that better captures racially unequal access to social, political, and economic resources. We used the Getis-Ord G-statistic (G_i*)[77,78] which addresses both challenges.[26] In MESA, G_i* is based on the racial composition of a census tract and a distance-decayed one-mile radius buffer around its centroid within the larger Core Based Statistical Area (CBSA), which is a Census Bureau-defined area containing ≥1 urbanized core along with adjacent counties with a high degree of social and economic integration (e.g., a metropolitan area). The census includes information on specific racial and ethnic groups. We focused on specific groups to match the racial/ethnic information in MESA. The 2000 census and MESA both include information on Black or White race and Hispanic ethnicity. We combined this information to create racial/ethnic groups that reflect potential sociopolitical inequities. From the census, we used information on Black race with or without Hispanic ethnicity (i.e., 'Black'); White race without Hispanic ethnicity (i.e., 'non-Hispanic White' or 'NHW' from here); and Hispanic ethnicity of any race (i.e., 'Hispanic'). The G_i* is a z-score with greater values representing greater clustering segregation of Black, NHW, or Hispanic residents. To create mutually exclusive groups in MESA while reflecting potential sociopolitical inequities, we used information on Black race without Hispanic ethnicity (i.e., 'non-Hispanic Black' or 'NHB' from here); White race without Hispanic ethnicity (i.e., 'NHW'); and Hispanic of any race (i.e., 'Hispanic'). Following the literature,[26,27,79] we matched the segregation measure at Exam 1 to the racial/ethnic category of the MESA participant. In addition to the continuous G_i*, we categorized measures based on critical values of the normal distribution corresponding to $p<0.05$ and $p<0.01$. We created categories that fit the distribution of MESA participants; for example, if cell sizes were too small for one of the racial/ethnic groups for the 'high clustered' group, then we did not use that category. Categories for NHB participants are: G_i*<1.96='no clustering'; G_i*≥1.96 and G_i*<2.58='clustering at the p<0.05 level'; and G_i*≥2.58='high clustering at the p<0.01 level'. Categories for NHW participants are: G_i*≤-1.96='underclustering at the p<0.05 level; G_i*>-1.96 and G_i*<1.96='no clustering', and G_i*≥1.96 as 'clustering at the p<0.05 level. Categories for Hispanic participants are: G_i*<1.96='no clustering' and G_i*≥1.96='clustering at the p<0.05 level'. Tract poverty data from the 2000 census was used to calculate the percent of persons below the poverty level at Exam 1. For regression models (but not descriptive characteristics), we standardardized the poverty measure for comparability to the segregation measure. We excluded participants without tract information (n=11).

Analytic approach

The distributions of continuous covariates were described using mean and standard deviation and categorical variables were described using median and interquartile range. Participants were excluded for missing demographic, tract, DNA methylation, or covariate data, or estimated monocyte proportions below the threshold (Supplemental Figure 2). We compared the excluded and analytic samples using t-tests for continuous variables and chi-square tests for categorical variables (Supplemental Table 1). All subsequent analyses were reported stratified by baseline self-reported race/ethnicity group due to little overlap in segregation values of NHB and NHW participants. When tract poverty was the focal exposure, we estimated models with all racial/ethnic groups together for consistency with the extant literature but also stratified by race/ethnicity, to provide information for the models that included poverty in the interaction term. Among included participants, the distributions of variables were reported, and we compared participants with low versus high DMAA. In bivariate analyses, we calculated the Pearson/Spearman correlation between DMAA and either segregation or poverty and visualized these associations with scatterplots.

For each clock, using ordinary least squares regression, we estimated models to evaluate the association of segregation, poverty, or their interaction, and DMAA stratified by race/ethnicity, excluding participants with missing covariate information (n=51) for a final analytic sample size of 1102 (DNA information was not collected on Chinese American participants because the site that included this group was not included in the MESA DNA methylation ancillary study). In Model 1, we tested for an association between segregation or poverty and DMAA without covariates. We then adjusted for the three cell type estimates with the largest

© 2023 Hicken MT et al. *JAMA Network Open.*

spread of values (i.e., monocytes, CD8 T lymphocytes, and B lymphocytes) and baseline self-reported gender (Model 2), important for precision. We further adjusted for the potential individual-level confounders of (Model 3) maternal education and one's own education, reported by the participant at Exam 1. We then adjusted for study site which might capture unmeasured confounders (Model 4A), but then removed it when adjusting for tract-level confounders (Model 4B) of poverty or segregation (whichever was not the focal exposure), measured at Exam 1. While study site may capture confounders between the tract-level exposure and outcomes, it may also be a driver of these exposures. Neighborhoods develop differently across the US; study site adjustment may remove the impact of those tract-level characteristics. To Model 4B, we included potential mediators (Model 5): smoking, alcohol use, BMI, and a count of chronic conditions, all measured at Exam 1. Information on smoking and alcohol use was collected through a series of questions on the consumption frequency and amount. Count of chronic condition was the sum of binary yes/no for self-report of the following conditions: cancer, arthritis, high blood pressure, kidney disease, diabetes, and hepatitis. BMI was calculated using height and weight measurements collected from MESA study staff. To account for multiple comparisons, we used the Benjamini-Hochberg correction to p-values.[80]

To examine the modifying role of tract poverty on the association between segregation and DMAA, we fit a model building upon model 4B with a term for the poverty-segregation multiplicative interaction. In all models we computed clustered standard errors based upon census tract. We report the regression coefficients, 95% confidence intervals (CI), and adjusted p-values for a one standard deviation increase in tract G_i^* score and for a standard deviation increase in tract poverty.

In sensitivity analyses, we estimated models with a subset of participants who were <55 years of age at baseline (N=377) as the MESA cohort was cardiovascular disease-free at their baseline ages of 45-84 and may have represented a particularly healthy group of adults, particularly at older ages. We also estimated models among only participants who did not move between 2000 and 2010 (N=834) to focus on those who may have had a more consistent neighborhood exposure over the ten-year follow-up period. (The sample size of movers was too small for analysis.) We estimated models using health and health behavior information from Exam 1, rather than Exam 5, in an attempt to adjust for factors that might be correlated with DNA methylation age clocks at baseline, since we do not have information on these clocks at Exam 1. We also estimated models using a categorical version of the segregation measures to reflect the statistical significance in clustering.

Analyses were performed in R statistical software (version 4.1.0). Code to produce all analyses and figures is available on GitHub (https://github.com/bakulskilab).

© 2023 Hicken MT et al. *JAMA Network Open.*

eTable 1. Participant characteristics of full, analytic, and excluded samples, Multi-Ethnic Study of Atherosclerosis (2000-2010)

	Full sample (n=1264)	Analytic sample[a] (n=1102)	Excluded sample (n=162)	p[b]
Tract segregation G_i*[c], z-score	1.96 (4.01)	1.86 (4.02)	2.70 (3.82)	n/a
Tract poverty, percentage[d]	0.17 (0.12)	0.16 (0.12)	0.19 (0.13)	0.046
Race/Ethnicity (%)				0.001
Non-Hispanic Black	22	20	32	
Non-Hispanic White	47	48	35	
Hispanic	32	32	33	
Women (%)	51	53		0.712
Age, years	69.6 (9.36)	69.7 (9.43)	68.7 (8.78)	0.167
Education, self (%)				0.220
≤High school	34	34	38	
>High school	28	27	31	
≥College	38	39	32	
Education, mother (%)				0.623
<High school	53	53	57	
=High school	29	29	27	
>High school	18	18	16	
Smoking status (%)				0.090
Current	9	8	14	
Former	51	51	51	
Never	40	41	36	
Never drank alcohol (%)	14	14	9	0.120
BMI, kg/m^2	29.3 (5.20)	29.1 (5.09)	30.0 (5.80)	0.075
Monocytes, percentage[e]	0.95 (0.05)	0.96 (0.02)	0.87 (0.09)	<0.001

Notes: Unless otherwise indicated, all values are mean (sd); percentages may not sum to 100 due to rounding. Chronological age, DNA methylation, and leukocyte type proportions were collected/measured at Exam 5; all other information was collected at Exam 1.

[a]Missing from the full sample: Gi*, 17; tract poverty, 11; education, self, 2; education, mother, 31; smoking status, 8; never drank alcohol, 2

[b]p-value for difference between analytic and excluded samples.

[c]Gi* tract segregation is calculated for non-Hispanic Black, non-Hispanic White, and Hispanic participants as the segregated clustering of Black, non-Hispanic White, and Hispanic residents, respectively. The average reported is across all racial/ethnic groups.

[d]Represents percentage of those in the tract living at or below the poverty level by tract of the individual participant's tract.

[e]Represents individual sample estimated monocyte proportions.

Abbreviations: BMI, body mass index; Gi*, Getis-Ord segregation index; n/a, no appropriate; sd, standard deviation

© 2023 Hicken MT et al. *JAMA Network Open.*

eTable 2. Interactive association among tract racial segregation, tract poverty, and DNA methylation age acceleration by race/ethnicity, Multi-Ethnic Study of Atherosclerosis (2000-2010)

	Non-Hispanic Black b (se)	Non-Hispanic White b (se)	Hispanic b (se)
GrimAge DNA methylation age acceleration			
Tract segregation	0.18 (0.11)	0.06 (0.12)	-0.04 (0.05)
Tract poverty	-0.03 (0.27)	0.70 (0.32)*	0.80 (0.33)*
Interaction	0.24 (0.09)**	0.13 (0.12)	-0.08 (0.04)*
Segregation-DNA methylation age association at:			
-1 sd poverty	-0.05 (0.17)	-0.07 (0.10)	0.04 (0.06)
mean poverty	0.18 (0.11)	0.06 (0.12)	-0.04 (0.05)
+1 sd poverty	0.42 (0.11)***	0.19 (0.22)	-0.12 (0.06)
Hannum DNA methylation age acceleration			
Tract segregation	-0.06 (0.12)	-0.16 (0.13)	0.07 (0.05)
Tract poverty	-0.34 (0.35)	-0.40 (0.49)	-0.31 (0.31)
Interaction	0.00 (0.11)	-0.17 (0.18)	-0.09 (0.05)
Segregation-DNA methylation age association at:			
-1 sd poverty	-0.06 (0.19)	0.00 (0.13)	0.16 (0.07)*
mean poverty	-0.06 (0.12)	-0.16 (0.13)	0.07 (0.05)
+1 sd poverty	-0.07 (0.14)	-0.33 (0.29)	-0.01 (0.08)
Horvath DNA methylation age acceleration			
Tract segregation	-0.08 (0.11)	-0.20 (0.10)*	0.04 (0.04)
Tract poverty	-0.73 (0.32)*	-0.60 (0.37)	-0.42 (0.26)
Interaction	0.06 (0.10)	-0.18 (0.12)	-0.01 (0.04)
Segregation-DNA methylation age association at:			
-1 sd poverty	-0.13 (0.17)	-0.02 (0.09)	0.05 (0.06)
mean poverty	-0.08 (0.11)	-0.20 (0.10)*	0.04 (0.04)
+1 sd poverty	-0.02 (0.12)	-0.38 (0.20)	0.03 (0.05)
PhenoAge DNA methylation age acceleration			
Tract segregation	0.34 (0.20)	0.02 (0.15)	0.07 (0.08)
Tract poverty	-0.51 (0.52)	-0.07 (0.57)	0.06 (0.51)
Interaction	0.03 (0.13)	0.20 (0.22)	-0.12 (0.07)
Segregation-DNA methylation age association at:			
-1 sd poverty	0.30 (0.28)	-0.19 (0.16)	0.19 (0.12)
mean poverty	0.34 (0.20)	0.02 (0.15)	0.07 (0.08)
+1 sd poverty	0.37 (0.18)*	0.22 (0.34)	-0.04 (0.10)

Notes: Gi* was calculated for non-Hispanic Black, non-Hispanic White, and Hispanic participants as the segregated clustering of Black, non-Hispanic White, and Hispanic residents, respectively. The association between segregation and DNA methylation age acceleration was calculated post-estimation from partial and interaction coefficients. Models were estimated with the following covariates: leukocyte type proportion (monocytes, CD8+, B cells), gender/sex, education-self, education-maternal.
*p<0.05; **p<0.01; ***p<0.001
Abbreviations: b, regression coefficient; se, standard error of the regression coefficient

© 2023 Hicken MT et al. *JAMA Network Open.*

eTable 3. Association between racial segregation and DNA methylation age acceleration by race/ethnicity, Multi-Ethnic Study of Atherosclerosis (2000-2010). Limited to those with baseline age <55.

Model	Non-Hispanic Black b (se)	Non-Hispanic White b (se)	Hispanic b (se)
GrimAge DNA methylation age acceleration			
1	0.32 (0.26)	-0.20 (0.11)	0.06 (0.06)
2	0.30 (0.25)	-0.29 (0.11)*	0.14 (0.06)*
3	0.20 (0.28)	-0.21 (0.14)	0.14 (0.06)*
4A	0.21 (0.28)	-0.32 (0.20)	0.14 (0.06)*
4B	0.13 (0.28)	-0.17 (0.15)	0.16 (0.07)*
5	-0.07 (0.30)	-0.04 (0.17)	0.12 (0.08)
Hannum DNA methylation age acceleration			
1	-0.10 (0.21)	-0.03 (0.11)	-0.10 (0.11)
2	0.01 (0.23)	-0.04 (0.11)	-0.05 (0.10)
3	-0.12 (0.23)	-0.05 (0.12)	-0.04 (0.11)
4A	-0.12 (0.23)	-0.06 (0.18)	-0.04 (0.09)
4B	-0.05 (0.25)	-0.08 (0.14)	0.04 (0.10)
5	-0.03 (0.26)	-0.08 (0.13)	0.04 (0.10)
Horvath DNA methylation age acceleration			
1	-0.26 (0.20)	0.05 (0.08)	-0.06 (0.07)
2	-0.18 (0.23)	0.07 (0.08)	-0.05 (0.07)
3	-0.29 (0.24)	0.05 (0.09)	-0.05 (0.08)
4A	-0.28 (0.24)	0.03 (0.11)	-0.04 (0.07)
4B	-0.14 (0.26)	-0.04 (0.09)	0.01 (0.07)
5	-0.07 (0.28)	-0.03 (0.09)	0.02 (0.06)
PhenoAge DNA methylation age acceleration			
1	0.33 (0.28)	-0.22 (0.19)	-0.05 (0.12)
2	0.31 (0.31)	-0.17 (0.18)	-0.08 (0.14)
3	0.21 (0.31)	-0.16 (0.21)	-0.06 (0.16)
4A	0.21 (0.31)	-0.16 (0.33)	-0.05 (0.15)
4B	0.25 (0.32)	-0.31 (0.20)	0.06 (0.17)
5	0.33 (0.32)	-0.26 (0.22)	0.07 (0.16)

Notes: Gi* was calculated for non-Hispanic Black, non-Hispanic White, and Hispanic participants as the segregated clustering of Black, non-Hispanic White, and Hispanic residents, respectively.

Models were estimated with the following covariates: (1) no covariates; (2) leukocyte type proportion (monocytes, CD8+, B cells), gender/sex; (3) Model 2 covariates plus education-self, education-maternal; (4A) Model 3 covariates plus site; (4B) Model 3 covariates plus tract poverty; (5) Model 4B covariates plus smoking status, never drank alcohol, body mass index, count of chronic conditions (sum of binary yes/no for self-report of the following conditions: cancer, arthritis, high blood pressure, kidney disease, diabetes, and hepatitis).

*p<0.05; **p<0.01; ***p<0.001

Abbreviations: b, regression coefficient; se, standard error of the regression coefficient

© 2023 Hicken MT et al. *JAMA Network Open.*

eTable 4. Association between tract poverty and DNA methylation age acceleration by race/ethnicity, Multi-Ethnic Study of Atherosclerosis (2000-2010). Limited to those with baseline age <55.

Model	Entire sample b (se)	Non-Hispanic Black b (se)	Non-Hispanic White b (se)	Hispanic b (se)
GrimAge DNA methylation age acceleration				
1	0.22 (0.21)	0.55 (0.46)	0.39 (0.56)	0.01 (0.25)
2	0.42 (0.21)*	0.76 (0.49)	0.63 (0.55)	0.15 (0.25)
3	0.41 (0.22)	0.65 (0.50)	0.55 (0.58)	0.09 (0.26)
4A	0.62 (0.24)*	0.90 (0.56)	0.42 (0.59)	0.60 (0.31)
4B	0.45 (0.24)	0.57 (0.47)	0.22 (0.66)	-0.19 (0.30)
5	0.43 (0.24)	0.73 (0.44)	0.09 (0.70)	-0.04 (0.32)
Hannum DNA methylation age acceleration				
1	-0.83 (0.20)***	-0.44 (0.44)	-0.06 (0.53)	-0.84 (0.29)**
2	-0.67 (0.21)**	-0.30 (0.48)	0.00 (0.55)	-0.66 (0.32)*
3	-0.63 (0.22)**	-0.55 (0.50)	0.00 (0.55)	-0.63 (0.29)*
4A	-0.47 (0.26)	-0.62 (0.56)	-0.12 (0.60)	-0.11 (0.41)
4B	-0.53 (0.25)*	-0.52 (0.53)	-0.15 (0.64)	-0.70 (0.34)*
5	-0.51 (0.26)	-0.48 (0.62)	-0.14 (0.66)	-0.62 (0.35)
Horvath DNA methylation age acceleration				
1	-0.71 (0.18)***	-0.74 (0.39)	-0.43 (0.28)	-0.49 (0.25)
2	-0.69 (0.18)***	-0.91 (0.42)*	-0.46 (0.27)	-0.47 (0.27)
3	-0.66 (0.19)***	-1.20 (0.48)*	-0.45 (0.27)	-0.44 (0.27)
4A	-0.47 (0.24)	-1.10 (0.52)*	-0.24 (0.26)	-0.38 (0.37)
4B	-0.62 (0.20)**	-1.10 (0.51)*	-0.53 (0.32)	-0.45 (0.26)
5	-0.62 (0.21)**	-1.20 (0.64)	-0.53 (0.34)	-0.52 (0.28)
PhenoAge DNA methylation age acceleration				
1	-0.46 (0.31)	-0.30 (0.56)	-0.01 (0.92)	-0.53 (0.40)
2	-0.57 (0.34)	-0.12 (0.56)	-0.20 (0.94)	-0.90 (0.44)*
3	-0.61 (0.36)	-0.18 (0.63)	-0.25 (0.99)	-0.87 (0.46)
4A	-0.58 (0.39)	-0.28 (0.69)	-0.58 (1.10)	-0.44 (0.57)
4B	-0.61 (0.37)	-0.34 (0.65)	-0.85 (1.10)	-0.97 (0.53)
5	-0.62 (0.38)	-0.45 (0.74)	-0.76 (1.10)	-1.00 (0.55)

Notes: Models were estimated with the following covariates: (1) no covariates; (2) leukocyte type proportion (monocytes, CD8+, B cells), gender/sex; (3) Model 2 covariates plus education-self, education-maternal (race/ethnicity is included in models using the entire analytic sample); (4A) Model 3 covariates plus site; (4B) Model 3 covariates plus racial segregation; (5) Model 4B covariates plus smoking status, never drank alcohol, body mass index, count of chronic conditions.

*p<0.05; **p<0.01; ***p<0.001

Abbreviations: b, regression coefficient; se, standard error of the regression coefficient

© 2023 Hicken MT et al. *JAMA Network Open.*

eTable 5. Association between racial segregation categories and DNA methylation age acceleration by race/ethnicity, Multi-Ethnic Study of Atherosclerosis (2000-2010)

Model	Non-Hispanic Black		Non-Hispanic White		Hispanic
	Clustering b (se)	High clustering b (se)	Under clustering b (se)	Clustering b (se)	Clustering b (se)
GrimAge DNA methylation age acceleration					
1	0.99 (0.65)	1.85 (0.60)**	0.70 (0.36)	-0.57 (0.54)	-0.05 (0.40)
2	1.15 (0.70)	1.57 (0.55)**	0.64 (0.39)	-0.63 (0.51)	0.31 (0.38)
3	1.18 (0.71)	1.60 (0.58)**	0.61 (0.36)	-0.32 (0.44)	0.31 (0.40)
4A	1.09 (0.79)	1.58 (0.59)**	0.67 (0.34)*	-0.28 (0.80)	0.35 (0.41)
4B	1.41 (0.68)*	0.96 (0.57)	0.35 (0.66)	-0.34 (0.44)	0.01 (0.44)
5	1.30 (0.65)*	0.68 (0.57)	0.07 (0.56)	-0.67 (0.41)	0.02 (0.44)
Hannum DNA methylation age acceleration					
1	-1.13 (1.01)	-0.81 (0.65)	0.42 (0.31)	-0.12 (0.83)	-1.00 (0.55)
2	-1.01 (0.91)	-0.88 (0.64)	0.41 (0.31)	-0.17 (0.83)	-0.74 (0.55)
3	-1.33 (0.93)	-1.21 (0.61)*	0.37 (0.32)	-0.02 (0.82)	-0.74 (0.56)
4A	-1.47 (0.93)	-1.21 (0.63)	0.71 (0.37)	1.03 (0.86)	-0.36 (0.47)
4B	-1.45 (0.92)	-0.88 (0.65)	0.76 (0.55)	0.01 (0.82)	-0.04 (0.61)
5	-1.46 (0.93)	-0.89 (0.66)	0.86 (0.55)	0.13 (0.82)	0.00 (0.62)
PhenoAge DNA methylation age acceleration					
1	0.20 (1.28)	1.17 (0.87)	-0.64 (0.40)	-1.82 (0.81)*	-0.46 (0.65)
2	0.22 (1.30)	1.27 (0.89)	-0.52 (0.44)	-1.63 (0.90)	-0.50 (0.66)
3	0.02 (1.45)	1.06 (0.94)	-0.49 (0.42)	-1.36 (0.89)	-0.39 (0.68)
4A	-0.23 (1.41)	1.04 (0.94)	-0.62 (0.43)	-0.87 (1.17)	-0.04 (0.70)
4B	-0.11 (1.45)	1.43 (1.02)	-0.49 (0.73)	-1.36 (0.89)	0.13 (0.80)
5	-0.07 (1.39)	1.48 (1.03)	-0.47 (0.71)	-1.28 (0.90)	0.38 (0.77)
Horvath DNA methylation age acceleration					
1	-1.20 (0.86)	-1.11 (0.57)	0.28 (0.23)	-0.71 (0.52)	-0.30 (0.35)
2	-1.07 (0.77)	-1.05 (0.57)	0.30 (0.23)	-0.63 (0.54)	-0.29 (0.35)
3	-1.15 (0.80)	-1.14 (0.60)	0.31 (0.22)	-0.60 (0.55)	-0.24 (0.34)
4A	-1.49 (0.81)	-1.15 (0.59)	0.15 (0.27)	-0.31 (0.78)	-0.09 (0.33)
4B	-1.36 (0.76)	-0.53 (0.61)	0.95 (0.37)**	-0.55 (0.54)	0.26 (0.32)
5	-1.40 (0.76)	-0.51 (0.64)	0.92 (0.37)*	-0.46 (0.52)	0.36 (0.32)

Notes: Gi* was calculated for non-Hispanic Black, non-Hispanic White, and Hispanic participants as the segregated clustering of Black, non-Hispanic White, and Hispanic residents, respectively. The referent group for all comparisons is ‘no clustering’.

Models were estimated with the following covariates: (1) no covariates; (2) leukocyte type proportion (monocytes, CD8+, B cells), gender/sex; (3) Model 2 covariates plus education-self, education-maternal; (4A) Model 3 covariates plus site; (4B) Model 3 covariates plus tract poverty; (5) Model 4B covariates plus smoking status, never drank alcohol, body mass index, count of chronic conditions (sum of binary yes/no for self-report of the following conditions: cancer, arthritis, high blood pressure, kidney disease, diabetes, and hepatitis).

*p<0.05; **p<0.01; ***p<0.001

Abbreviations: b, regression coefficient; se, standard error of the regression coefficient

© 2023 Hicken MT et al. *JAMA Network Open.*

eTable 6. Association between racial segregation and DNA methylation age acceleration by race/ethnicity, Multi-Ethnic Study of Atherosclerosis (2000-2010). Limited to those who did not change census tracts between Exam 1 and Exam 5

Model	Non-Hispanic Black b (se)	Non-Hispanic White b (se)	Hispanic b (se)
**GrimAge DNA methylation age acceleration**			
1	0.43 (0.11)***	-0.13 (0.10)	-0.09 (0.05)
2	0.35 (0.09)***	-0.13 (0.10)	-0.03 (0.05)
3	0.38 (0.09)***	-0.09 (0.10)	-0.04 (0.05)
4A	0.38 (0.09)***	-0.07 (0.10)	-0.03 (0.05)
4B	0.30 (0.10)**	-0.03 (0.12)	-0.09 (0.06)
5	0.25 (0.09)**	-0.03 (0.10)	-0.09 (0.06)
**Hannum DNA methylation age acceleration**			
Model	Non-Hispanic Black b (se)	Non-Hispanic White b (se)	Hispanic b (se)
1	0.03 (0.12)	-0.08 (0.08)	-0.12 (0.08)
2	-0.01 (0.12)	-0.07 (0.09)	-0.09 (0.08)
3	-0.06 (0.12)	-0.04 (0.08)	-0.08 (0.08)
4A	-0.06 (0.12)	0.00 (0.09)	-0.05 (0.05)
4B	0.06 (0.14)	0.00 (0.10)	0.04 (0.07)
5	0.06 (0.15)	0.00 (0.10)	0.04 (0.07)
**Horvath DNA methylation age acceleration**			
Model	Non-Hispanic Black b (se)	Non-Hispanic White b (se)	Hispanic b (se)
1	-0.04 (0.11)	-0.02 (0.06)	-0.04 (0.05)
2	-0.04 (0.11)	-0.01 (0.06)	-0.04 (0.05)
3	-0.06 (0.12)	0.00 (0.06)	-0.03 (0.05)
4A	-0.06 (0.12)	0.06 (0.07)	-0.02 (0.04)
4B	0.09 (0.12)	-0.03 (0.08)	0.05 (0.04)
5	0.11 (0.13)	-0.02 (0.08)	0.05 (0.04)
**PhenoAge DNA methylation age acceleration**			
Model	Non-Hispanic Black b (se)	Non-Hispanic White b (se)	Hispanic b (se)
1	0.44 (0.17)**	-0.04 (0.11)	0.01 (0.08)
2	0.44 (0.17)**	-0.03 (0.12)	0.01 (0.09)
3	0.41 (0.19)*	0.00 (0.12)	0.05 (0.09)
4A	0.41 (0.19)*	0.13 (0.15)	0.07 (0.10)
4B	0.55 (0.21)**	-0.10 (0.15)	0.09 (0.10)
5	0.57 (0.21)**	-0.09 (0.15)	0.10 (0.09)

Notes: Gi* was calculated for non-Hispanic Black, non-Hispanic White, and Hispanic participants as the segregated clustering of Black, non-Hispanic White, and Hispanic residents, respectively.
Models were estimated with the following covariates: (1) no covariates; (2) leukocyte type proportion (monocytes, CD8+, B cells), gender/sex; (3) Model 2 covariates plus education-self, education-maternal; (4A) Model 3 covariates plus site; (4B) Model 3 covariates plus tract poverty; (5) Model 4B covariates plus smoking status, never drank alcohol, body mass index, count of chronic conditions (sum of binary yes/no for self-report of the following conditions: cancer, arthritis, high blood pressure, kidney disease, diabetes, and hepatitis).
*p<0.05; **p<0.01; ***p<0.001
Abbreviations: b, regression coefficient; se, standard error of the regression coefficient

© 2023 Hicken MT et al. *JAMA Network Open.*

eTable 7. Association between tract poverty and DNA methylation age acceleration by race/ethnicity, Multi-Ethnic Study of Atherosclerosis (2000-2010). Limited to those who did not change tracts between Exam 1 and Exam 5

Model	Entire sample b (se)	Non-Hispanic Black b (se)	Non-Hispanic White b (se)	Hispanic b (se)
GrimAge DNA methylation age acceleration				
1	0.45 (0.13)***	0.73 (0.27)**	0.47 (0.28)	0.20 (0.18)
2	0.51 (0.12)***	0.79 (0.24)***	0.48 (0.29)	0.27 (0.16)
3	0.46 (0.13)***	0.75 (0.24)**	0.48 (0.26)	0.28 (0.16)
4A	0.66 (0.14)***	0.99 (0.24)***	0.49 (0.24)*	0.62 (0.25)**
4B	0.48 (0.13)***	0.48 (0.25)*	0.45 (0.28)	0.40 (0.20)*
5	0.47 (0.12)***	0.61 (0.22)**	0.28 (0.28)	0.41 (0.20)*
Hannum DNA methylation age acceleration				
Model	Entire sample b (se)	Non-Hispanic Black b (se)	Non-Hispanic White b (se)	Hispanic b (se)
1	-0.66 (0.14)***	-0.38 (0.27)	0.09 (0.27)	-0.61 (0.21)**
2	-0.59 (0.14)***	-0.29 (0.27)	0.10 (0.26)	-0.58 (0.21)**
3	-0.41 (0.15)**	-0.44 (0.28)	0.08 (0.26)	-0.61 (0.20)**
4A	-0.16 (0.16)	-0.43 (0.29)	0.15 (0.28)	0.22 (0.25)
4B	-0.38 (0.15)**	-0.39 (0.30)	-0.09 (0.33)	-0.73 (0.20)***
5	-0.36 (0.15)*	-0.38 (0.32)	-0.06 (0.33)	-0.69 (0.22)**
Horvath DNA methylation age acceleration				
Model	Entire sample b (se)	Non-Hispanic Black b (se)	Non-Hispanic White b (se)	Hispanic b (se)
1	-0.42 (0.10)***	-0.67 (0.21)**	-0.04 (0.21)	-0.35 (0.16)*
2	-0.40 (0.10)***	-0.61 (0.22)**	-0.03 (0.20)	-0.38 (0.16)*
3	-0.40 (0.11)***	-0.68 (0.23)**	-0.03 (0.20)	-0.37 (0.16)*
4A	-0.26 (0.12)*	-0.58 (0.26)*	-0.09 (0.19)	-0.18 (0.22)
4B	-0.38 (0.11)***	-0.65 (0.24)**	-0.24 (0.25)	-0.46 (0.15)**
5	-0.38 (0.11)***	-0.70 (0.25)**	-0.21 (0.25)	-0.52 (0.16)**
PhenoAge DNA methylation age acceleration				
Model	Entire sample b (se)	Non-Hispanic Black b (se)	Non-Hispanic White b (se)	Hispanic b (se)
1	-0.31 (0.16)*	-0.21 (0.32)	-0.23 (0.32)	-0.43 (0.27)
2	-0.27 (0.17)	-0.09 (0.33)	-0.19 (0.33)	-0.48 (0.28)
3	-0.30 (0.19)	-0.13 (0.34)	-0.20 (0.32)	-0.44 (0.30)
4A	-0.01 (0.21)	-0.01 (0.36)	-0.17 (0.34)	0.41 (0.41)
4B	-0.31 (0.19)	-0.43 (0.37)	-0.43 (0.40)	-0.53 (0.33)
5	-0.30 (0.19)	-0.42 (0.36)	-0.38 (0.40)	-0.68 (0.32)*

Notes: Models were estimated with the following covariates: (1) no covariates; (2) leukocyte type proportion (monocytes, CD8+, B cells), gender/sex; (3) Model 2 covariates plus education-self, education-maternal (race/ethnicity is included in models using the entire analytic sample); (4A) Model 3 covariates plus site; (4B) Model 3 covariates plus racial segregation; (5) Model 4B covariates plus smoking status, never drank alcohol, body mass index, count of chronic conditions.

*p<0.05; **p<0.01; ***p<0.001

Abbreviations: b, regression coefficient; se, standard error of the regression coefficient

© 2023 Hicken MT et al. *JAMA Network Open.*

eFigure 1. GrimAge DNA methylation age acceleration as raw and residual values as a function of chronological age, in years, Multi-Ethnic Study of Atherosclerosis (2010)

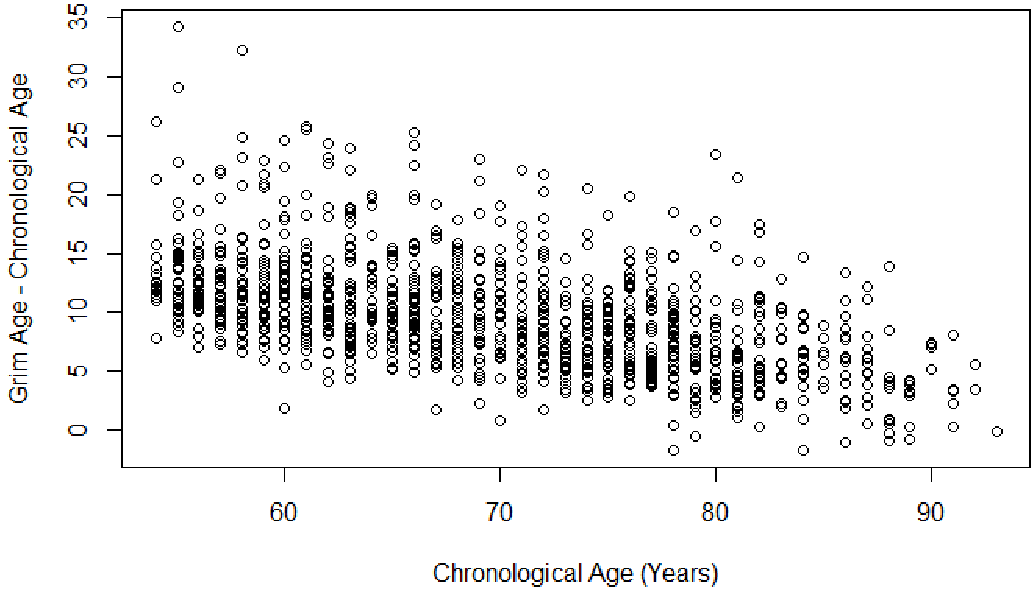

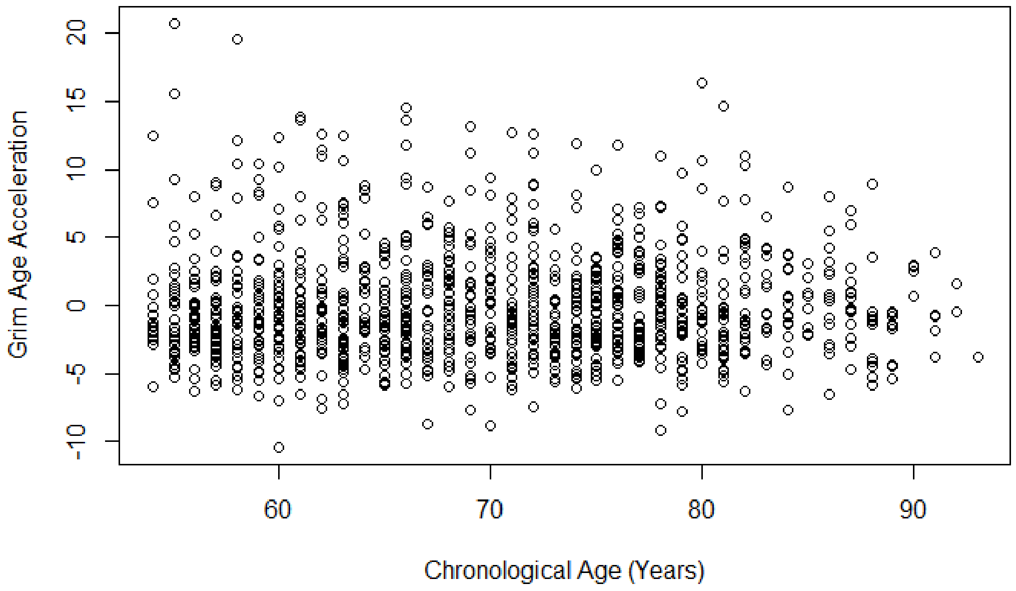

Notes: GrimAge-Chronological Age is the raw difference between the two measures. GrimAge Acceleration is the residual from the regression of chrological age on GrimAge.

© 2023 Hicken MT et al. *JAMA Network Open.*

eFigure 2. Flow chart of inclusion into analytic sample

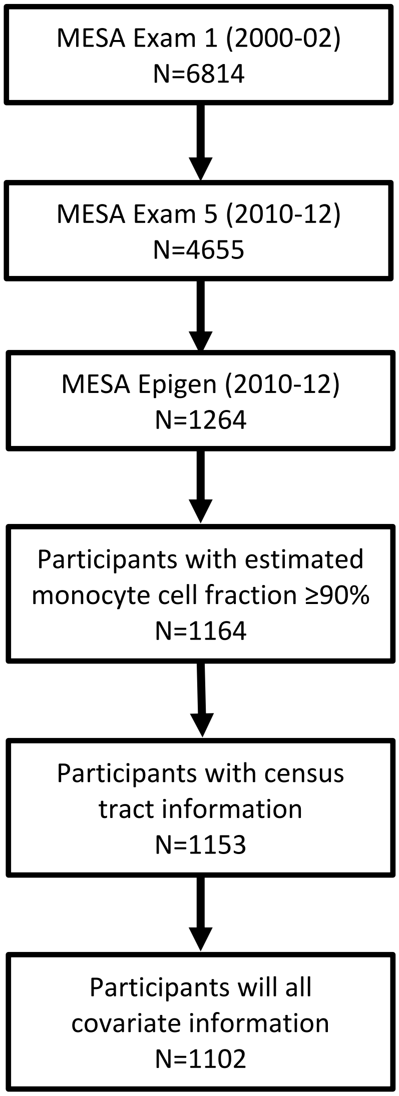

© 2023 Hicken MT et al. *JAMA Network Open.*

eFigure 3. Racial segregation and tract poverty, by race/ethnicity, Multi-Ethnic Study of Atherosclerosis (2000-2010)

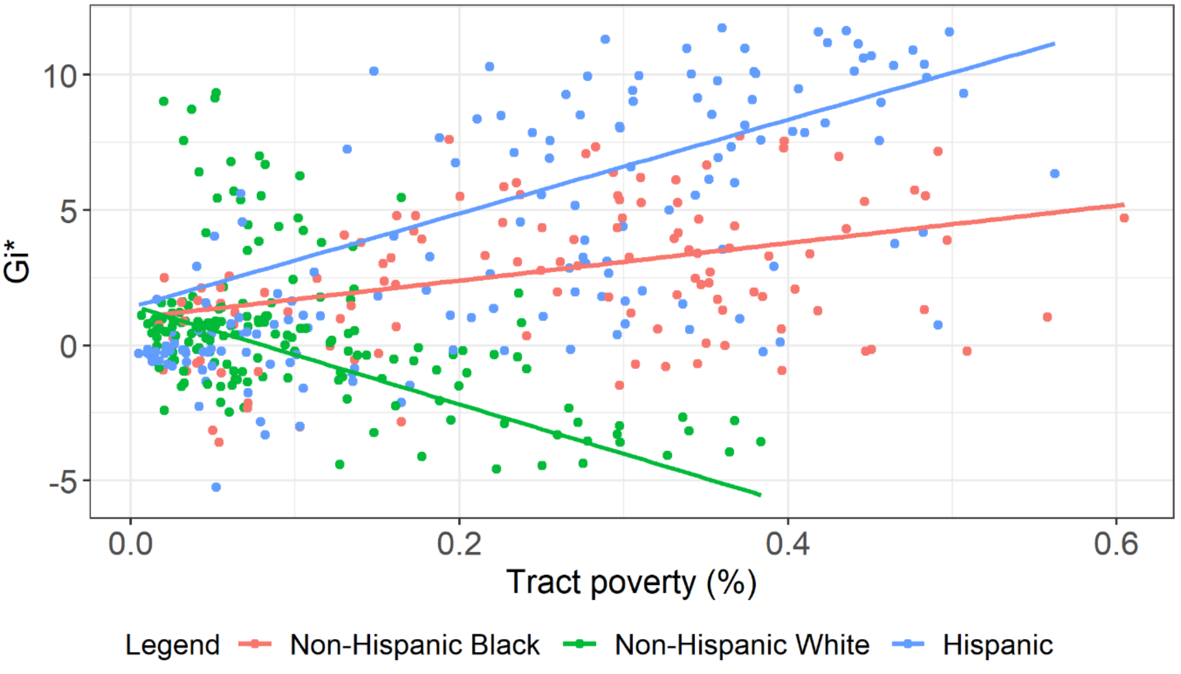

Notes: Gi* is calculated for non-Hispanic Black, non-Hispanic White, and Hispanic participants as the segregated clustering of Black, non-Hispanic White, and Hispanic residents, respectively.

© 2023 Hicken MT et al. *JAMA Network Open.*

eFigure 4. Hannum, Horvath, and PhenoAge DNA methylation age acceleration as a function of racial segregation by race/ethnicity and tract poverty, Multi-Ethnic Study of Atherosclerosis (2000-2010)

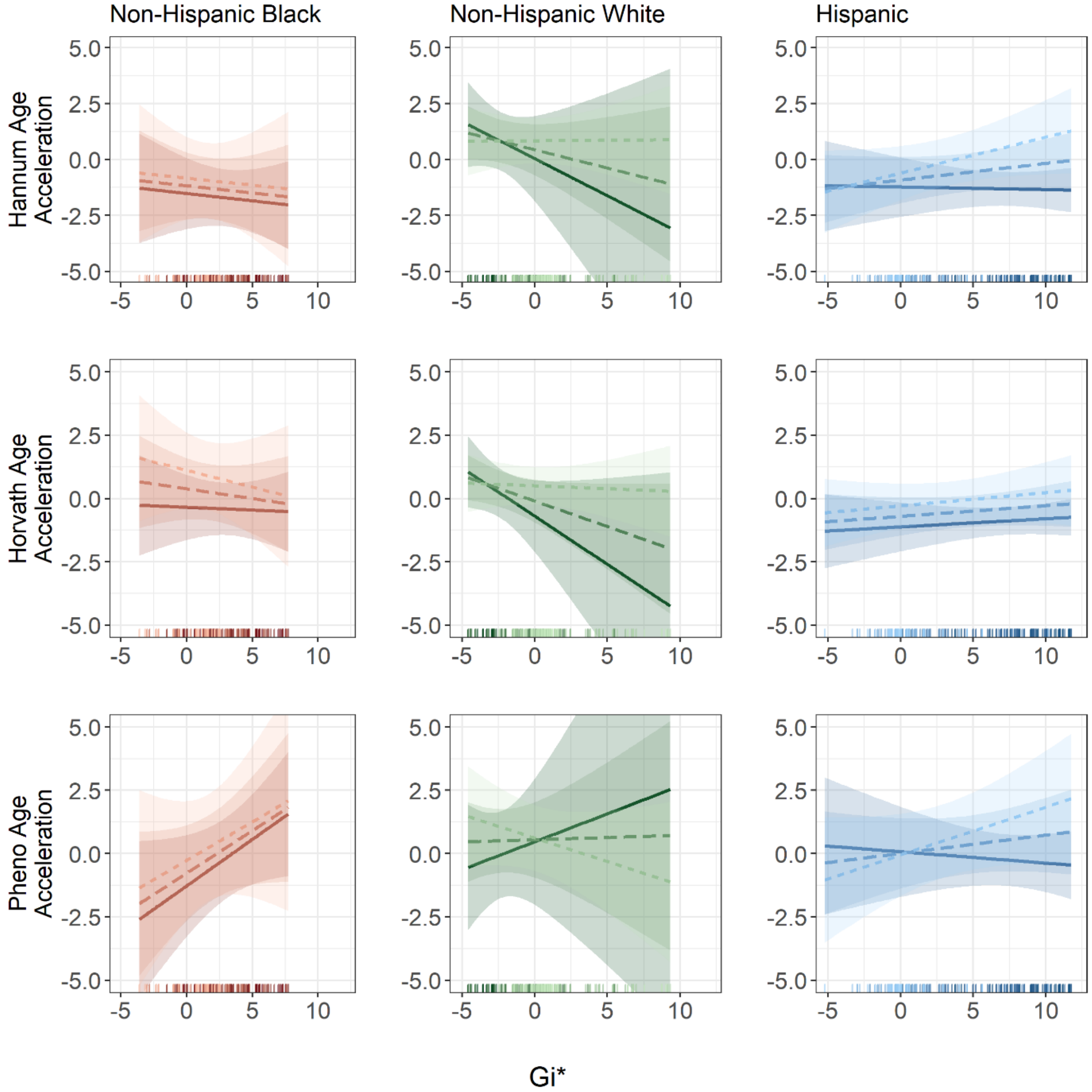

Legend: — 1 standard deviation above mean tract poverty
– – mean tract poverty
····· 1 standard deviation below mean tract poverty

Notes: Gi* is calculated for non-Hispanic White, non-Hispanic Black, and Hispanic participants as the segregated clustering of non-Hispanic White, Black, and Hispanic residents, respectively. Models were estimated with the following covariates: leukocyte type proportion, gender/sex, education-self, education-maternal

© 2023 Hicken MT et al. *JAMA Network Open.*
