# Supplementary material for: Racial and Ethnic Residential Segregation and Monocyte DNA Methylation Age Acceleration
Source: JAMA Netw Open. 2023 Nov 29;6(11):e2344722. doi: 10.1001/jamanetworkopen.2023.44722 (PMC10687663; doi:10.1001/jamanetworkopen.2023.44722)
Supplement: Supplement 2. — Data Sharing Statement [file jamanetwopen-e2344722-s002.pdf]

Data Sharing Statement

Hicken. Racial and Ethnic Residential Segregation and Monocyte DNA Methylation Age Acceleration. *JAMA Netw Open*. Published November 29, 2023.
doi:10.1001/jamanetworkopen.2023.44722

Data
Data available: Yes
Data types: Deidentified participant data
How to access data: MESA data are available by contacting the core center at https://www.mesa-nhlbi.org/Publications.aspx.
When available: With publication

Supporting Documents
Document types: Statistical/analytic code
How to access documents: We will provide the code via GitHub.
When available: With publication

Additional Information
Who can access the data: Link to code will be available to article readers.
Types of analyses: The code for all analyses in the published article will be available to readers.
Mechanisms of data availability: Data will be made available by request, by and at the discretion of the MESA Core Center.
